# Paraborg: Paramedic Cyborg Insects for In Situ Emergency Drug‐Delivery

**DOI:** 10.1002/advs.76973

**Published:** 2026-08-05

**Authors:** H. Nhan Le, P. Thien Phan, Lachlan Fitzgerald, P. Ross McAree, T. Nho Do, T. Thang Vo‐Doan

**Affiliations:** ^1^ School of Mechanical and Mining Engineering The University of Queensland Brisbane Australia; ^2^ School of Biomedical Engineering Faculty of Engineering Tyree Institute of Health Engineering University of New South Wales Sydney Australia

**Keywords:** biohybrid robots, cooperative teleoperation, cyborg insects, drug‐delivery, macropanesthia rhinoceros, paramedic robot

## Abstract

Over the past two decades, cyborg insects have emerged as a promising alternative to artificial insect‐scale mobile robots, owing to their low power consumption, resilient biological bodies with naturally integrated sensors, agile locomotion, and self‐adaptability. However, most studies have focused primarily on autonomous control and hardware integration for search‐oriented urban search‐and‐rescue operations following natural disasters such as earthquakes. This raises a crucial next‐step challenge: beyond locating survivors, what life‐saving interventions can cyborg insects perform? Here, we present paramedic cyborg insects (Paraborgs) equipped with a human‐supervised auto‐injection mechanism (AIM) and onboard camera, enabling needle‐based in situ liquid‐drug‐delivery executed by multiple teleoperated cyborg cockroaches. The feasibility of Paraborg is demonstrated through three core innovations: (i) a compact auto‐injection mechanism that launches an injector and delivers liquid drug to a target with an 90% success rate; a robust manual control strategy achieving 100% success in reaching designated checkpoints, 72% success in stabilizing cyborg cockroaches for targeted injection, and up to 95% close‐range injection success within 150 mm of the target; and (iii) teamwork concept with multiple teleoperated cyborg insects for carrying out complex in situ functions. Together, these results demonstrate Paraborg as a prospective platform that extends cyborg insect swarm toward field‐deployable medical intervention.

## Graphical abstract

This work introduces Paraborg, a human‐supervised paramedic cyborg‐insect platform that combines controllable locomotion, onboard imaging, and an auto‐injection mechanism for in situ drug‐delivery. By demonstrating reliable navigation, target stabilization, injector launch, and multi‐insect teamwork, the study extends cyborg insects from survivor detection toward active medical intervention in confined post‐disaster environments.

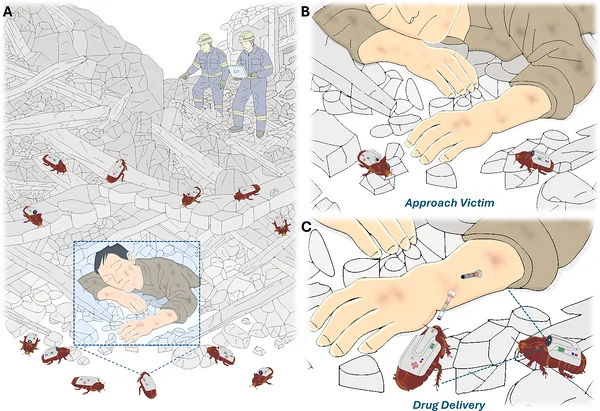

## 1 Introduction

Each year, dozens of major earthquakes worldwide cause widespread building collapses, creating hazardous environments that severely hinder urban search and rescue (USAR) operations [1, 2]. The resulting debris fields and unstable structures not only complicate access but also delay rescue efforts, often leading to reduced survival rates among trapped victims. For instance, the 7.8‐magnitude earthquake that struck Turkey on 6 February 2023 affected an area of 350 000 km^2^, requiring the deployment of USAR teams from 91 countries to locate victims trapped beneath the debris [3, 4]. Financial records also show that hundreds of millions of dollars in grants and pledges were allocated in response to the catastrophe, including support for USAR operations [5]. These statistics highlight the urgent need to develop efficient strategies for search‐and‐rescue missions during and after major natural disasters. In remote and challenging environments, such as mountainous or otherwise hard‐to‐reach areas, even delays of minutes to hours in accessing medical assistance can lead directly to preventable deaths or lifelong disabilities [6, 7]. Conditions like snakebite envenomation and postpartum hemorrhage illustrate this vulnerability: both require immediate administration of lifesaving injections such as antivenom or oxytocin, yet timely treatment is often hindered by the difficulty of traversing rugged terrains. Similar urgency exists in cases of severe allergic reactions, sepsis, or trauma, where survival depends on the rapid delivery of injectable therapeutics. These realities underscore the urgent need for agile, highly maneuverable delivery systems capable of traversing narrow, debris‐filled spaces that often block direct access to trapped victims and hinder the provision of timely emergency care.

Drones and legged mobile robots can be deployed in the aforementioned emergency scenarios [8, 9, 10]; however, in environments with complex terrain and victims buried beneath collapsed structures, their size and limited locomotion capabilities often prevent them from accessing narrow spaces to effectively locate trapped individuals. To address this challenge, miniature or soft robots have been proposed [11, 12, 13, 14, 15, 16, 17, 18]; yet the current state of artificial components, such as chassis materials [19, 20, 21], centimeter‐scale actuators and sensors [22, 23], and energy supply [24, 25], still falls short of enabling small‐scale mobile robots with low power consumption, high fatigue durability, and the self‐adaptability required for navigating in complex terrain (Table S1). As an alternative, cyborg insects, i.e., a biohybrid mobile robot platform that leverages the organic structures of living insects [26, 27, 28, 29], have emerged as a promising candidate to overcome these limitations for practical USAR operations [30]. These biohybrid robots offer two intrinsic advantages: (i) robust biological exoskeletons for mechanical strength [31, 32, 33, 34], and (ii) naturally integrated sensors and self‐adaptive behavior enabled by their central nervous systems [35, 36, 37, 38, 39]. Furthermore, they significantly reduce fabrication costs and energy demands compared to fully artificial miniature robots (Table S1). Over the past two decades, cyborg insects have been successfully demonstrated in various USAR‐related tasks, such as human detection and obstacle avoidance using onboard cameras [40, 41, 42], wide‐area exploration through coordinated cyborg swarm [43], sound localization to navigate toward trapped victims [44, 45], autonomous operation through feedback control [46, 47, 48], improved locomotion via artificial component integration [49], and self‐powered systems to extend operational duration [50, 51]. However, despite these advances, the functional capabilities of cyborg insects have remained largely within the domain of “search” rather than “rescue”. In large‐scale disaster scenarios, the ability to provide immediate medical assessment and basic stabilization can significantly improve outcomes for victims awaiting rescue. However, current USAR systems offer limited capacity for medical intervention within confined spaces, while existing remote drug‐delivery (RDD) approaches lack the mobility required to operate effectively in complex disaster environments.

Currently, RDD is still conducted manually to perform the “shot” and then injection effectively [52], while the latest autonomous injecting technologies have been focusing on non‐invasive oral drug‐delivery, i.e., these systems attempt to provide medicines—vaccines through endoscopic procedures [53, 54, 55, 56, 57] or ingestible capsules [58, 59, 60, 61, 62, 63, 64]. Nevertheless, on‐field USAR after natural catastrophes requires both mobile drug‐delivery and autonomous functionalities due to the restricted operational space and unconscious patients. Needle‐based RDD mechanisms, similar to anesthesia or dart guns, can be considered as an available option [65, 66, 67]; however, their bulky characteristic and strong penetrating force at short distances will be inconvenient and potentially dangerous during search and rescue missions [65, 66, 68]. Although existing autonomous drug‐delivery principles have been developed and verified meticulously to achieve cogent reliability (e.g., insertion‐force characterization, self‐orientation capability, self‐activation), they still lack the mobility, proactive triggering, and rapid‐injecting process to be equipped for USAR mobile robots.

In this study, we present an in situ, needle‐based drug‐delivery system performed by teleoperated paramedic cyborg insects (Paraborgs, Figure 1) to assist trapped and unconscious victims in emergency scenarios, achieving ∼95% success at target distances within 150 mm. While prior research on terrestrial cyborg insects has largely focused on basic locomotion control, such as left/right turning [69, 70, 71, 72, 73], forward movement [47, 69, 71, 74], and induced immobility [70], this work introduces advanced strategies tailored for medical intervention tasks. These include a U‐turn counter to overcome non‐responsive motion under antennal stimulation and a combined set of control rules that stabilize Paraborgs during drug‐delivery, reaching up to 100% success in checkpoint arrival and target injection. In addition, we describe the design, fabrication, and experimental validation of a compact auto‐injection mechanism (AIM), integrated with a printed circuit board (PCB), which achieved a 90% success rate and enabled liquid delivery once the Paraborg was stabilized. Two key capabilities are demonstrated: (i) successful in situ drug‐delivery using AIM under real‐time supervision via an onboard camera, and (ii) coordinated navigation by two Paraborgs in a co‐located setting. Together, these findings establish the feasibility of future human‐supervised cyborg swarms in which multiple paramedic insects, each equipped with specialized medical functions, can be deployed in real‐world rescue operations.

**FIGURE 1:**
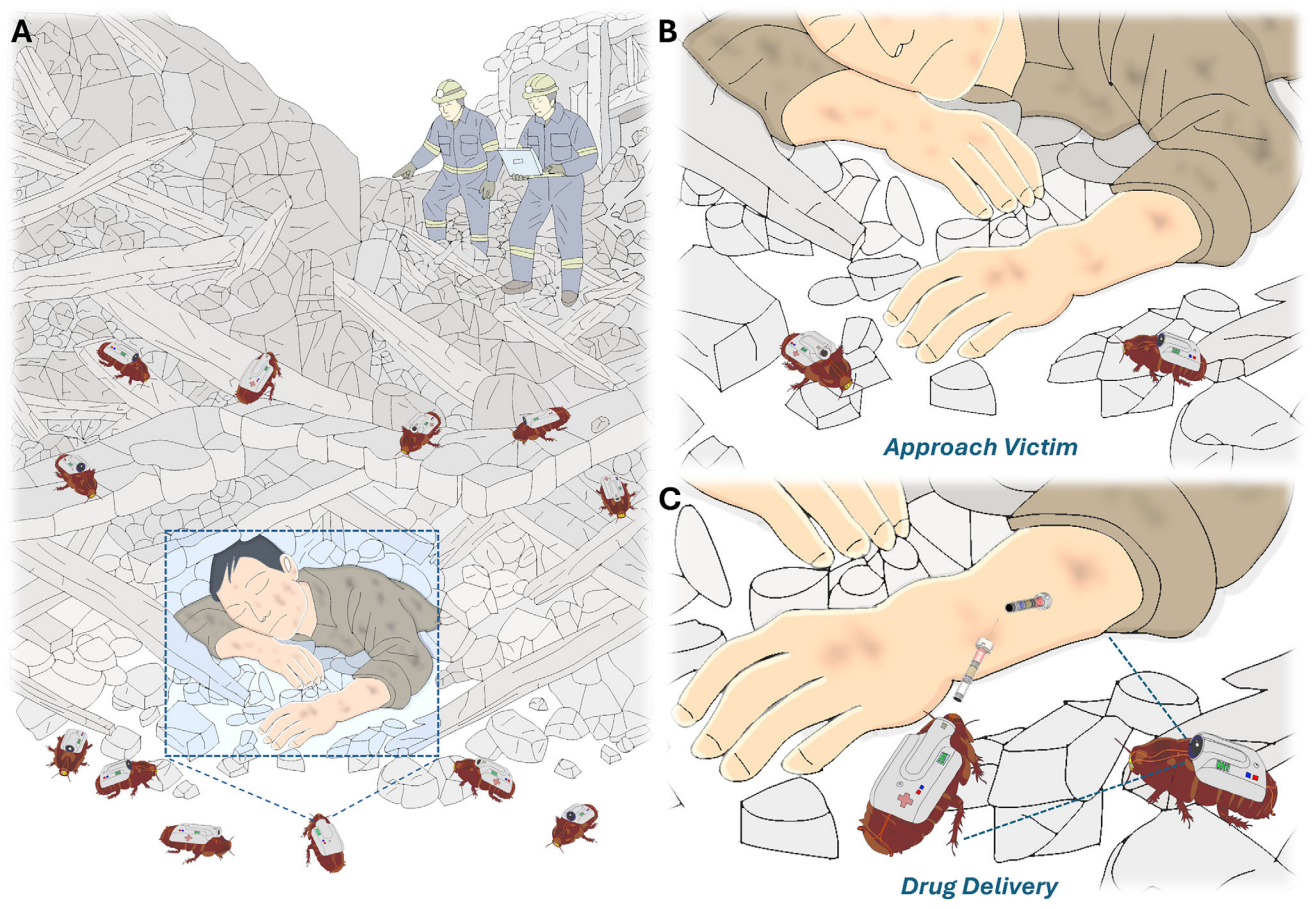
Tentative scenario for real‐world deployment of Paraborgs. (A) A human‐supervised swarm of paramedic cyborg cockroaches is deployed in a complex disaster environment for victim localization and on‐site medical assistance. (B) After victim identification, two cyborg cockroaches, equipped with a teleoperated auto‐injection mechanism (left) and an onboard camera (right), navigate through collapsed structures to locate victims. (C) Once the camera‐carrying cyborg locates the victim and the drug‐delivery cyborg is positioned and immobilized, the injection mechanism is remotely triggered. The injector launches and delivers a liquid drug in situ to help stabilize the victim's condition until rescue.

## 2 Results

### 2.1 Human‐in‐the‐Loop Teleoperation of Multi‐Paraborg

The deployment of Paraborgs equipped with AIM for on‐field drug‐delivery operations will require integration with onboard sensors to monitor the human‐supervised launching and auto‐injection processes, particularly in post‐earthquake emergency scenarios where victims may be trapped beneath collapsed structures (Figure 1). Additional onboard cameras can be considered as one of the most available approaches at the moment, as they possess compact size [42, 75, 76] and strong implementation of a human recognition system [40, 41, 46]. However, integrating multiple hardware modules such as AIM and an onboard camera onto a single Paraborg imposes trade‐offs. These include increased energy consumption, which limits the wireless operational time, and added weight and bulk, which can impair the insect's mobility and effectiveness in navigating complex terrains. To address these limitations and leverage recent advancements in cyborg swarm systems [43, 77], this study introduces a teleoperated teamwork‐based approach utilizing at least two Paraborgs to perform complex in situ operations. In this configuration, one Paraborg, designated as the “observer”, was equipped with a wireless Wi‐Fi camera to assist in victim search and drug‐delivery monitoring (Figure 1). This observer Paraborg provides real‐time images to the main control computer, enabling emergency responders to manipulate the position and injector launching by the drug‐delivery Paraborg (Figure 1).

Although the field of cyborg insects has advanced rapidly toward autonomy, extending beyond reliance on onboard sensors to include multi‐agent swarm systems, practical deployment in search‐and‐rescue scenarios also requires in situ intervention capabilities, including drug‐delivery. For trapped victims awaiting rescue, timely first‐aid support may be critical to survival. In such high‐stakes contexts, human‐supervised teleoperation represents a more appropriate near‐term strategy than full autonomy. In particular, the final decision to initiate first‐aid protocols, such as drug‐delivery, should remain with trained medical personnel and experienced first responders during search‐and‐rescue missions, rather than relying solely on cyborg insects as mobile autonomous search robots guided entirely by integrated onboard sensors, as seen in existing studies. In this work, Paraborg with AIM is designed to serve as an advanced first‐aid tool for search‐and‐rescue teams, while also establishing a foundation for the future development of practical autonomous functions that support human operators. This human‐in‐the‐loop approach enables operator supervision of critical intervention tasks, such as injection, while complementing autonomous search functions that do not directly intervene in the victim's body.

### 2.2 Controllability of Paraborg via Electrical Stimulation

By using adjusted parameters for electrical stimulation at antennae and both cerci, graded turning and accelerating responses were achieved for Paraborgs with *Macropanesthia rhinoceros* platform (Figure 2, Figures S1–S5, and Movie S1), which can potentially support the implementation of closed‐loop control systems [47]. Notably, the locomotion performance of Paraborgs equipped with the integrated hardware was comparable to that of natural giant burrowing cockroaches (Figure S6 and Movies S2 and S3). When varying applied frequency in the range 10–50 Hz, average induced turning angle by stimulation at unilateral antenna demonstrated a stable increase from 27.7 to 59.5 degrees for left antenna and 15.5 degrees to 45.1 degrees for right antenna (one‐sample *t*‐test, *n* = 5 cockroaches, *n* = 125 stimuli per stimulation site, *t* (24) > 5 for each applied frequency value, *p* < 0.001, p‐values were adjusted using Bonferroni correction, Figure 2D and Figure S5). This indicates a graded relationship of ∼0.86 deg/Hz for left antenna stimulation and ∼0.75 deg/Hz for right antenna stimulation (Linear regression, Spearman's Correlation, *n* = 5 cockroaches, *n* = 125 stimuli, *p* < 0.05, *ρ* > 0.44, Figure 2D and Figure S5).

**FIGURE 2:**
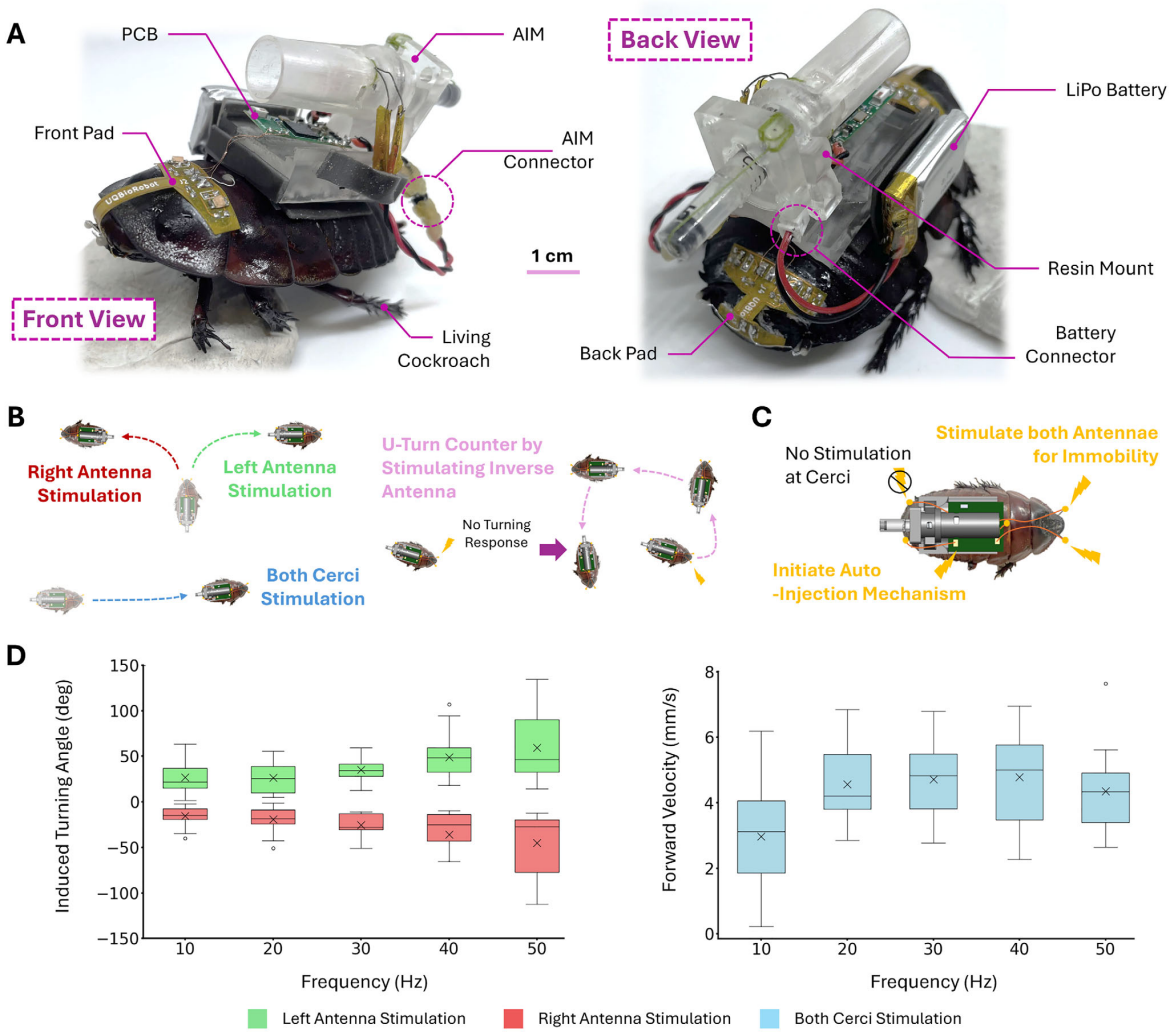
Locomotion performance of paramedic cyborg cockroaches. (A) Overview of the terrestrial cyborg cockroach and its integrated hardware. The paramedic cockroach is equipped with compact components, including an AIM module for in situ drug‐delivery, flexible pads and a PCB for electrical stimulation, and a LiPo battery powering both the AIM and PCB for wireless operation. (B) Locomotion control strategies with electrical stimulation. The paramedic cyborg cockroach can perform basic turning and acceleration maneuvers like a mobile robot, with a “U‐turn counter” implemented to preserve controllability under response deterioration in a unilateral antenna. (C) Drug‐delivery control strategy. A combination of control rules, i.e., electrical stimulation of the antenna and cerci alongside AIM triggering, is applied to stabilize the cockroach during the injector‐launching process. (D) Motor responses of the giant burrowing cockroach to electrical stimulation at a unilateral antenna and both cerci. Experimental data are presented as boxplots with 1.5 × IQR whiskers; crosses indicate mean values.

Although applying higher frequency to both cerci also led to greater accelerating response ∼0.03 mms^−1^Hz^−1^ (Linear regression, Spearman's Correlation, *n* = 5 cockroaches, *n* = 125 stimuli, *p* < 0.05, *ρ* = ∼0.2, Figure 2D and Figure S5), the relationship between the applied stimulation frequency at both cerci and the resulting acceleration response does not exhibit a graded response in the giant burrowing cockroach, given the weak correlation observed; especially in the frequency range 20–40 Hz, the maximum difference in average forward velocity was merely lower than 1 mm/s (one‐sample *t*‐test, *n* = 5 cockroaches, *n* = 125 stimuli, *t* (24) > 7 for each applied frequency value, *p* < 0.001, p‐values were adjusted using Bonferroni correction, Figure 2D and Figure S5). Furthermore, the behavior of Paraborgs under 50 Hz cerci stimulation implies over‐responsiveness [78], i.e., the cockroach accelerated rapidly after the initiation of stimulation but illustrated a significant downtrend in forward velocity after 150 ms (Figure 2D and Figure S5). Therefore, the 10–40 Hz range was a more suitable frequency range for cerci stimulation of giant burrowing cockroaches. On the other hand, fixing the value of applied frequency, which is similar to the earlier study [47], will be also a rational strategy for this insect platform.

### 2.3 Auto‐Injection Mechanism (AIM) for In Situ Needle‐Based Drug‐Delivery

Most existing studies have reported that the maximum load‐carrying capacity of cockroaches without impairing locomotion is approximately 1–2 times their body weight [79, 80]. Accordingly, given a body weight of ∼34–40 g, a practical constraint can be established such that the total weight of integrated components should remain below ∼50 g (i.e., ∼1.5 times the body weight of *Macropanesthia rhinoceros*), ensuring that their terrestrial locomotion is preserved. To satisfy this design constraint, AIM was designed to be compact and lightweight and was validated to have no significant effect on cockroach locomotion (Figures S6 and S7 and Movie S2), i.e., a ready‐to‐use set, including AIM module, resin mount, PCB, other electronic components, only increases the total height and weight of a living cockroach 15 mm and ∼17 g respectively (Table S2). The injection can be executed by triggering an electrical signal (∼2 A, ∼3.5 s duration) from the PCB. The drug‐delivery function of this module occurs with two consecutive steps: launching and injecting. For the launching, the injector is pushed into the launch tube to compress a spring (8 mm diameter, 23 mm length, Figure 3A and Figure S8). This injector, which has a collar with a fishing line near its needle, is then fixed at a specific position, i.e., where the spring is compressed maximally and held by tying a knot of the line to the holes on the cylinder tube (Figure 3A). Finally, a Nichrome (NiCr) wire (0.3 mm diameter, 40 mm length) is threaded through the knot and connected to the PCB terminal. When the triggering electrical current is applied, the temperature of the NiCr wire increases by resistance heating and cuts the fishing line to release the spring (Figure 3A–C). The spring force pushes the inner injector out of the launch tube. For injecting, this step starts automatically after the injector leaves the launch tube from the previous launching process. The second fishing line, which penetrates through a membrane isolating citric acid (C_6_H_8_O_7_) in the behind chamber and potassium bicarbonate powder (KHCO_3_) in the front syringe cylinder (Figure 3B), is pulled back and breaks the membrane as soon as the injector leaves the launch tube completely (Figure 3C and Figures S8 and S9). The two isolated chemicals are then mixed and react, generating carbon dioxide (CO_2_) and thus increasing pressure inside the syringe cylinder. This pressure presses the rubber stopper to inject the medicine into the target (Figure 3B,C, Figures S8 and S9 and Movie S3).

**FIGURE 3:**
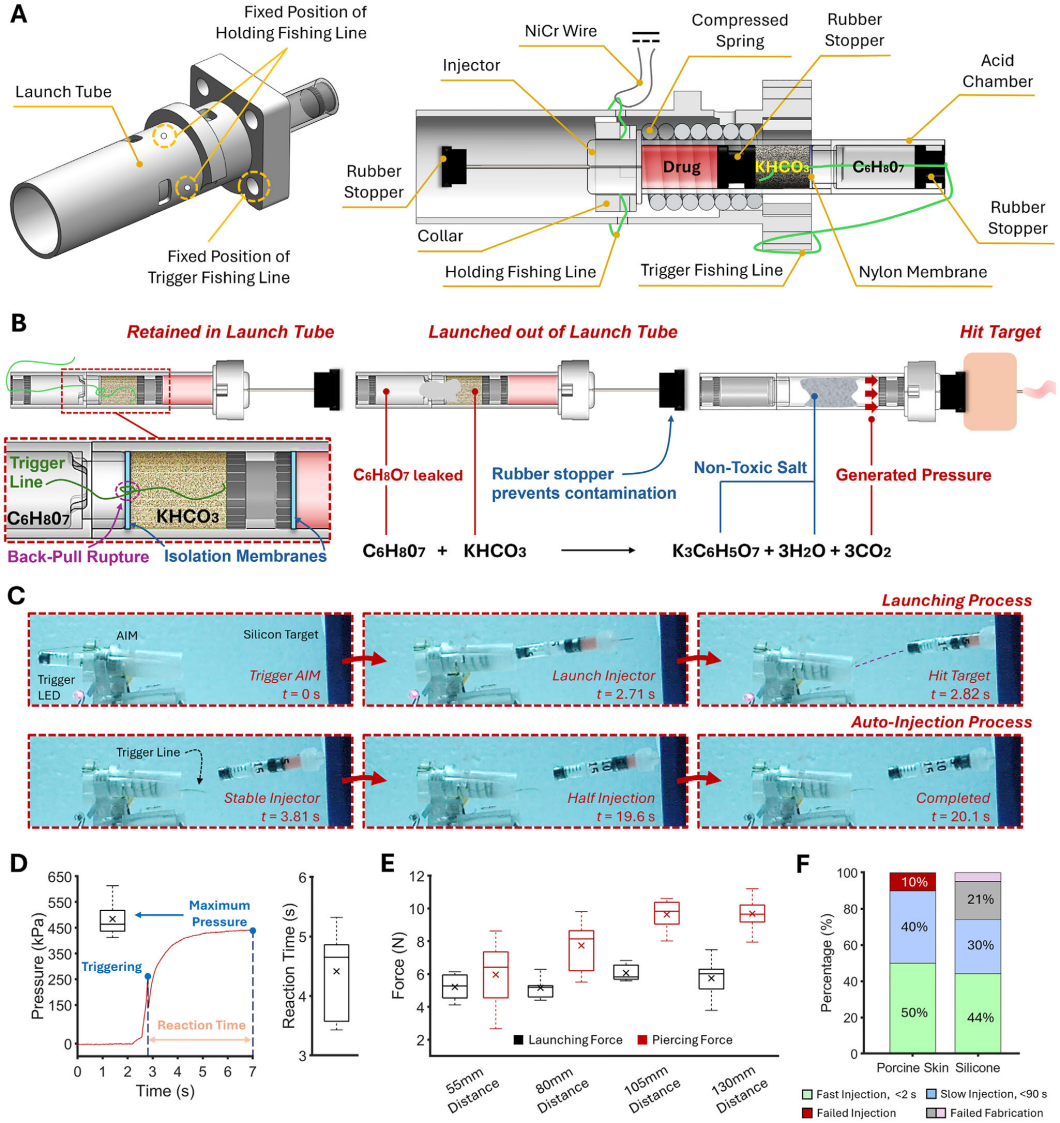
Development of the auto‐injection mechanism (AIM). (A) Mechanical design overview. (B, C) Auto‐injection principle. The AIM operates via a chemical reaction between citric acid and potassium bicarbonate powders, stored separately in the rear and front syringe cylinders. Upon launch from the tube, the trigger line retracts, releasing liquid acid into the front chamber. The reaction between the two chemicals generates pressure, driving the rubber stopper to inject the liquid drug through the needle. (D) Verification of the chemical reaction. Pressure sensors measure the peak generated pressure and reaction time, enabling adjustment of chemical quantities for a safe and reliable injection. (E) Verification of forces during launch. Launching force and piercing force upon impact with a silicone target are analyzed. Experimental data are shown as boxplots with 1.5 × IQR whiskers; crosses indicate mean values. (F) AIM performance in ex vivo launch‐and‐injection trials. Penetration and autonomous drug‐delivery efficiency were evaluated using porcine skin and silicone targets (10 trials each). Five AIM units were repeatedly tested to assess reusability until failure, defined as chemical leakage causing premature injection or launch‐tube fracture leading to unsuccessful deployment. Detailed reuse data are provided in Table S3.

For the pressure generated by the chemical reaction, preliminary calculations indicate that the reaction can rapidly increase the internal pressure to approximately 314 kPa, producing an estimated 2.2‐N injection force (detailed calculations are provided in the Supporting Information). When the reaction proceeds longer, experimental measurements using the pressure‐sensor setup (Figure S9) show that the reaction between C_6_H_8_O_7_ and KHCO_3_ requires approximately 4.5 s on average to reach its peak pressure of ∼470 kPa (Figure 3D).

To validate the efficiency of the spring mechanism for launching and safety factors when the injector penetrates through the human skin, launching and piercing forces are analyzed from the measured data from two load cells (Figure S9). Average launching force measured by the left loadcell demonstrates the repulsion by compressed spring to the injector varying in a stable range ∼5.6–6 N (*n* = 4 launching distances, *n* = 40 trials, one‐sample *t*‐test, *t* (9) > 4 per launching distance, *p* < 0.05, Figure 3E), which aligns closely with the estimated ∼5.2 N spring force reported in the Supporting Information. By this repulsion force, the injector guarantees a 100% successful rate in accurately hitting an 80 × 100‐mm silicone target. On the other hand, experiment results of the piercing force illustrate a transparent trend that further launching distance leads to stronger penetration when the injector contacts the target, i.e., average piercing forces of distances 55 – 80 – 105 – 130 mm are ∼6, ∼7.9, ∼9.8, and ∼9.9 N respectively (*n* = 40 trials, *n* = 4 launching distances, one‐sample *t*‐test, *p* < 0.05, *t* (9) > 3 per launching distance, Figure 3E). To ensure the required safe force for needle‐assisted jet injections, distances shorter than 50–55 mm will be the preferred effective launch range for AIM when it can provide decent output force and stable injection; while longer distances up to 130 mm can still be applied in actual field operation.

When equipped for paramedic cyborg cockroaches, AIM is triggered by an electrical signal from the PCB backpack with 3.3 V amplitude (∼6.6 W power) within ∼3.5 s duration. To accommodate the high current required for AIM activation, the ignition circuit is electrically isolated from electrical stimulation and wireless communication circuitry through an N‐channel MOSFET (Figure S9D). While the amplitude was determined based on the technical specifications of the battery and wireless PCB, the duration of this triggering signal is determined by considering the heating time of the NiCr wire to successfully cut the holding fishing line (Figure 3A) and launch the injector. Using a fixed resin mount positioned 50 mm from the target and a complete AIM system activated by a DC power supply (Figure S9C), experiments conducted across five AIM sets showed that each set could be reused 3–4 times (Table S3). In all trials, the chemical reaction and subsequent pressure generation required less than approximately 2 min to deliver the liquid drug through the needle when targeting silicone (74% success rate, ∼5.5 mm penetration depth, Figure 3F and Movie S3) and less than approximately 1.5 min when targeting porcine skin (90% success rate, ∼5.5 mm penetration depth, Figure 3F and Movie S3). Preliminary contamination testing further confirmed no detectable drug contamination resulting from the chemical actuation process (Figure S10). Additionally, one porcine‐skin trial resulted in a failed injection because the injector followed a curved trajectory and missed the target entirely (Figure 3F, “Failed Injection” case). For the silicone‐target experiments, the “Failed Fabrication” cases were primarily attributed to accidental rupture of the separating membrane during assembly, which allowed acid to leak into the front syringe chamber. This unintended mixing initiated premature pressure generation, causing displacement of the rubber stopper before launch. Another observed failure mode was injector jamming, which occurred when the trigger fishing line failed to fully disengage from the acid chamber and became trapped behind the rubber stopper. This issue was associated with structural degradation of reused launch tubes, which exhibited rebound‐induced fracture after repeated use. Although syringe cylinders were intentionally reused in these experiments to evaluate reusability, we recommend employing a new syringe set for each real‐world deployment to ensure hygiene and reliability.

### 2.4 Navigation and Drug‐Delivery With Wieldy Manual Control System

In this study, the capability of Paraborgs to carry and trigger the onboard AIM was validated in an experimental arena (Figure 4A and Figure S11). Paraborgs were required to navigate through three checkpoints before entering an effective launch range (Figure 4 and Figure S11). After passing checkpoint 3, the primary objective was to stabilize the cockroach in alignment with the injection target and initiate the in situ drug‐delivery function. Utilizing a reliable manual remote‐control with Bluetooth communication (Figure 4A and Figure S11A), Paraborgs with AIM demonstrated strong potential for real‐world deployment, where emergency responders could operate the paramedic cyborg insects in practical scenarios. The manual control system is designed based on four navigational states of the Paraborg: (i) walking toward the closest checkpoint, (ii) being immobile while oriented toward the checkpoint, (iii) exhibiting a heading angle deviation, or (iv) reaching checkpoint 3 (Figure 4B). Similar to prior control strategies for terrestrial cyborg insects [47, 72], unilateral antenna stimulation was applied when the insect deviated from the desired heading, and both cerci stimulation was used to overcome immobility. Conversely, Paraborgs were allowed to walk freely when oriented correctly toward the next checkpoint. Once Paraborg reached checkpoint 3 and entered the launch range, cerci stimulation was discontinued, and only low‐frequency (10–20 Hz) unilateral antenna stimulation was permitted. This approach aimed to reduce forward velocity and produce slight yaw adjustment until the insect aligned directly with the injection target (Figure 5 and Figure S12). Finally, bilateral antenna stimulation was applied to stabilize the cockroach during the triggering of the AIM system, completing the drug‐delivery function (Figure 4B).

**FIGURE 4:**
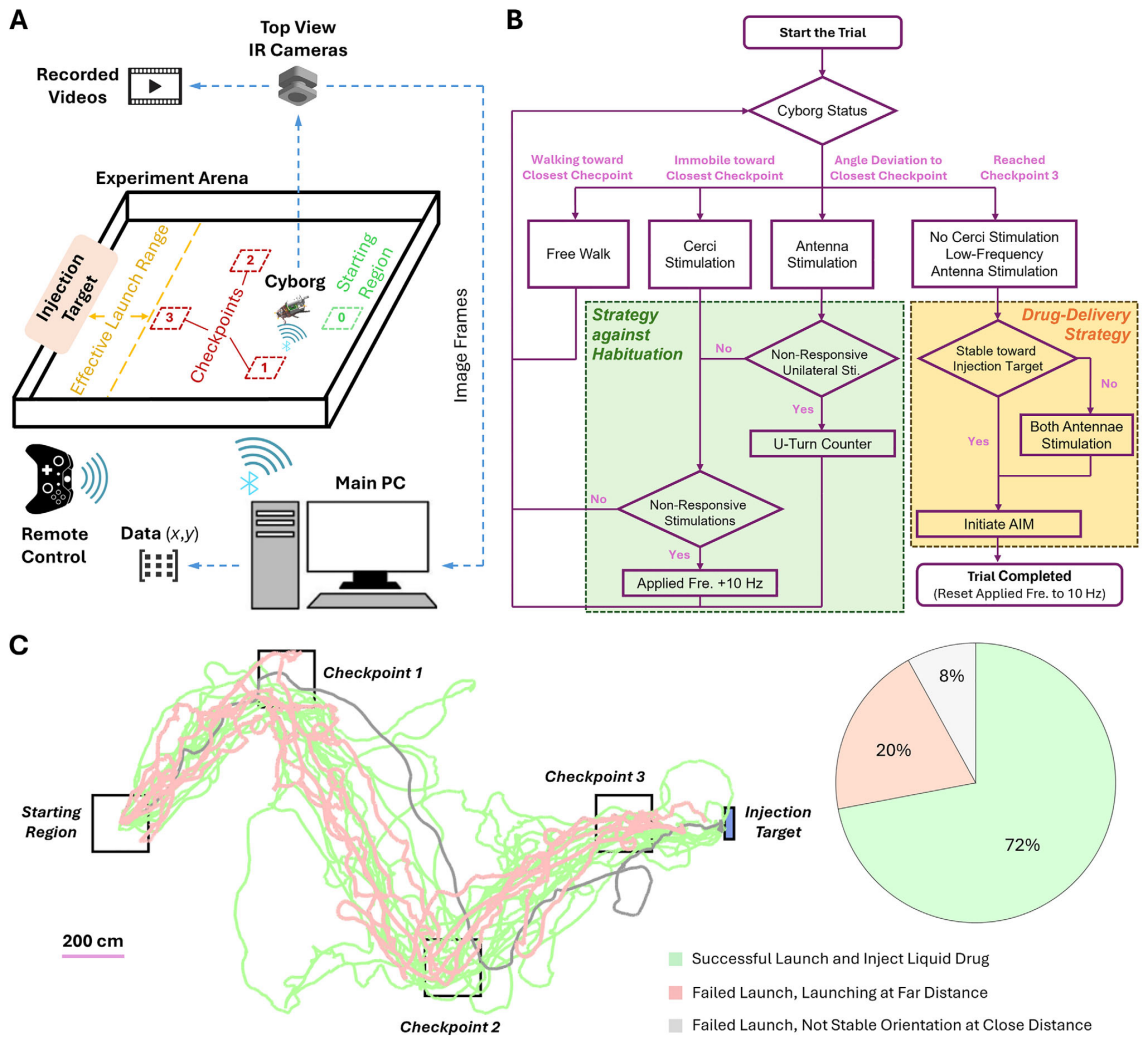
Performance of paramedic cyborg cockroaches in in situ drug‐delivery. (A) Experimental validation of locomotion and drug‐delivery capabilities. Paraborgs are required to reach three checkpoints before entering the effective launch range and performing successful in situ drug‐delivery with the AIM under remote manual control. Locomotion is monitored via top‐view cameras, providing both instantaneous position data (*x*, *y*) and corresponding video recordings. (B) Manual control strategies. The flow chart illustrates four operational states of the cockroach in the experimental arena: (i) walking toward the nearest checkpoint, (ii) immobile en route to the nearest checkpoint, (iii) deviating in heading from the checkpoint, and (iv) passing checkpoint 3. Two additional strategies for overcoming habituation and executing the drug‐delivery procedure are represented as green and yellow blocks, respectively. (C) Experimental outcomes of in situ drug‐delivery. Trials and corresponding tracked trajectories are categorized into three cases: (i) successful drug‐delivery after reaching all three checkpoints, (ii) reaching all three checkpoints but failing to launch the injector to hit the silicone target at far distances (∼150–250 mm), and (iii) reaching all three checkpoints but having the launched injector strike the wall instead of the silicone target due to sudden turning behavior after AIM activation.

**FIGURE 5:**
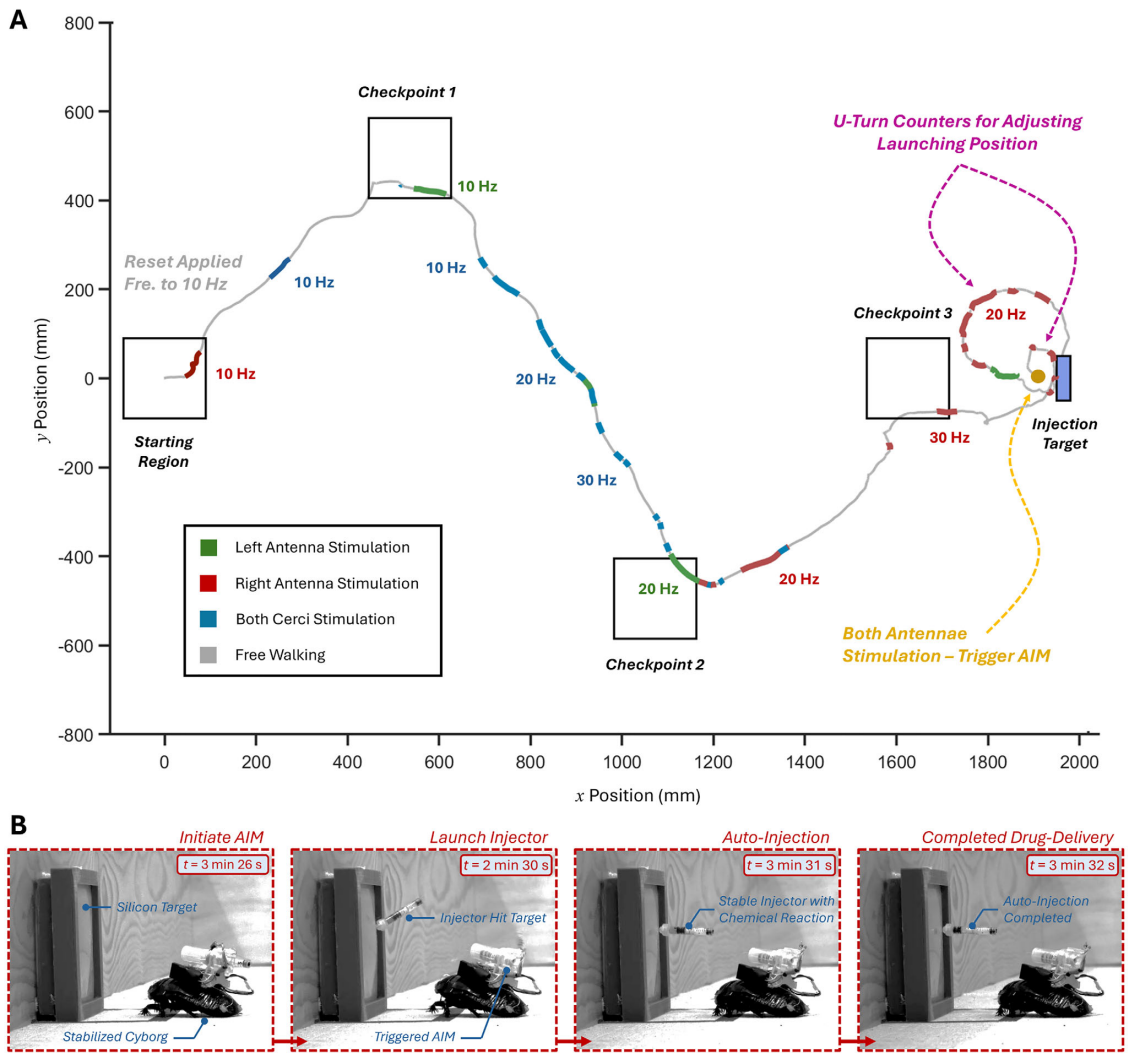
Representative trial of successful in situ drug‐delivery by single paramedic cyborg cockroach. (A) Demonstration of Paraborg locomotion controllability. Using the manual control strategies described in Figure 4B, the cyborg cockroach successfully reached three checkpoints and performed in situ drug‐delivery with the AIM, despite habituation occurring after checkpoint 1 and positional misalignment upon entering the effective launch range. (B) Demonstration of injector launch and automatic liquid delivery. Paraborg was stabilized at ∼25 mm from the silicone target before launching the injector, exhibiting only minor recoil (a detailed recoil analysis is presented in Figure S12). After piercing the silicone target, injector vibration ceased after ∼1 min, and the auto‐injection was completed within 1 s.

The reliability of this approach was comprehensively validated: only 2 out of 25 trials (8%) failed due to unexpected cockroach turning during the launch, which caused the injector to strike the wall instead of the target (Figure 4C). In contrast, the in situ drug‐delivery function showed higher success rates at shorter distances. Specifically, 72% of the trials (18/25) successfully launched and auto‐injected liquid into a silicone target (Figure 4C and Movie S4). Among these, 6 injections occurred at a distance of ∼50–150 mm, while the remaining 12 were triggered upon direct contact between the cockroach's head and the silicone layer (Figure 6C). However, failures were more frequent when launching from ∼150 to 250 mm, with 5 trials (20%) failing due to excessive piercing force causing the injector to rebound off the surface instead of penetrating it. This outcome suggests that the drug‐delivery function of Paraborg can achieve up to ∼95% success when triggered at close range, within 150 mm of the target.

**FIGURE 6:**
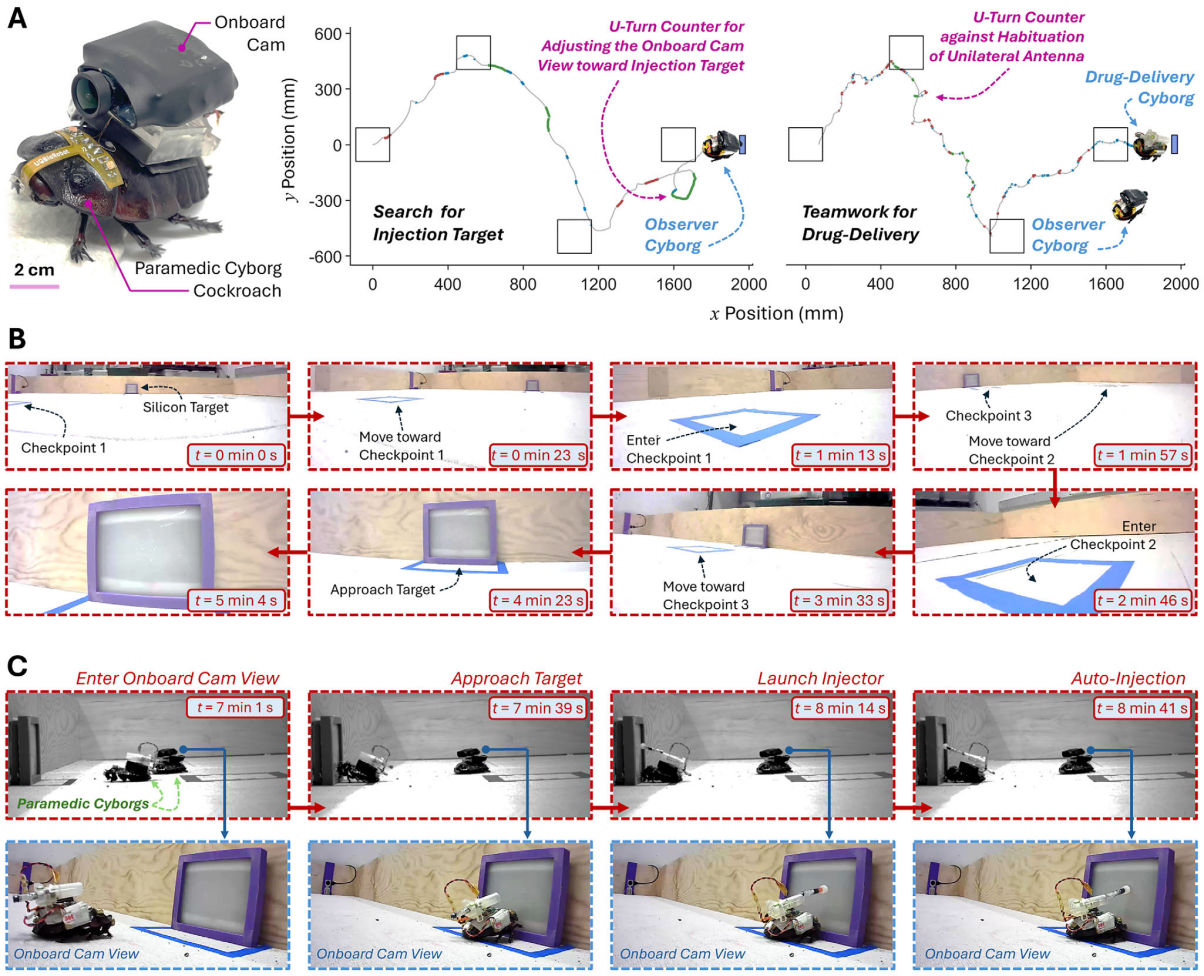
Teamwork concept with multiple teleoperated paramedic cyborg insects. (A) Support from an observer cyborg for on‐field deployment. A paramedic cyborg cockroach equipped with an onboard camera successfully navigated three checkpoints and approached the injection target, simulating a practical scenario in which an emergency responder team remotely operates the cyborg to traverse complex environments and locate trapped victims. After identifying the victim, a second teleoperated Paraborg equipped with the AIM followed the observer and triggered the injector, with the entire process monitored via real‐time visualization from the onboard Wi‐Fi camera. (B) Real‐time visual feed from the observer cyborg. The camera view is shown for key periods when the observer Paraborg entered checkpoints and approached the target. (C) Deployment of in situ drug‐delivery by two Paraborgs. Following the observer, the second cyborg entered the onboard camera's view, allowing the human operator to guide it toward the silicone target and launch the injector.

In addition to the drug‐delivery strategy, the manual control system of Paraborgs incorporated a dedicated anti‐habituation strategy (Figures 4B, 5A), designed to mitigate reduced responsiveness to electrical stimulation at the unilateral antenna, both antennae, and cerci. For cases of habituation at a unilateral antenna, a “U‐turn counter” was employed. This involved delivering multiple low‐frequency stimulations (10–20 Hz, 3–4 stimuli) to the contralateral antenna to elicit a U‐turn behavior (Figures 2B, 5A and Movies S4 and S5), thereby recovering the intended directional response. In more severe cases where the cockroach exhibited habituation to both antennae and cerci stimulation, the applied frequency was increased by 10 Hz for the affected sensory organs (Figures 4B, 5A and Movies S3–S7). This escalation aimed to overcome response deterioration and re‐establish responsiveness to control inputs. Using this habituation‐mitigation strategy, Paraborgs were successfully navigated in all experimental trials, achieving a 100% success rate in passing all three checkpoints and entering the effective launch range eventually (Figures 4C, 5 and Movies S4 and S6).

### 2.5 Teleoperated Teamwork Concept for Future Deployment

To achieve in situ drug‐delivery in emergency scenarios, deployment requires at least two teleoperated Paraborgs in the field, i.e., an observer and a drug‐delivery cyborg (Figure 1). During the process of searching for and subsequently delivering drugs to an unconscious victim, two typical field situations can be considered: (i) Paraborgs equipped with AIM and onboard cameras are dispersed when the observer cyborg detects a victim (Figure 6 and Movies S5 and S6), and (ii) both cyborgs are co‐located while searching for the trapped victim (Movie S7). In the first scenario, once the observer Paraborg successfully identifies a target with its onboard camera (Figure 6 and Movie S5), the strategy is for the second Paraborg carrying AIM to navigate toward the observer's location and enter the camera's field of view (Figure 6 and Movie S6). To enable this strategy, an IMU‐based (inertial measurement unit based) approach for continuous localization of multiple cyborgs, which is already demonstrated as feasible in existing studies [81], can be implemented to track the real‐time positions of both Paraborgs. Once the observer cyborg recognizes the victim, it will be stabilized and defined as the destination for the drug‐delivery cyborg. Finally, a target‐following algorithm can be implemented to guide the AIM‐carrying Paraborg toward the observer for in situ drug‐delivery (Movie S6) [47, 81].

For the second scenario, when two Paraborgs are co‐located before and after detecting a human victim, the camera‐equipped Paraborg serves not only as an observer providing real‐time visualization for emergency responders (Figure 6 and Movie S5), but also as a navigator and supervisor for other cyborg agents. By leveraging its onboard camera, the observer Paraborg can navigate in unknown environments while simultaneously supervising and coordinating nearby Paraborgs within its field of view, which is feasible through the dual‐agent demonstration in Movie S7. When visual contact was temporarily lost between two Paraborgs in narrow or debris‐filled environments, the front Paraborg (i.e., carrying AIM) can be stabilized at a position while the rear Paraborg (i.e., carrying an onboard camera) is stimulated to steer around the obstruction and performs a brief search to re‐establish visual contact (Movie S7). Once the front Paraborg re‐entered the camera field of view, dual‐agent navigation can be resumed. This approach has the potential to be enhanced further through employing IMU for localization or swarm navigation [43, 81], as well as infrared cameras combined with machine learning classifiers for human detection or obstacle avoidance [40, 41]. The demonstration presented in this study, i.e., navigating two Paraborgs using an onboard camera with the observer Paraborg acting as a third‐person monitor (Figure 7 and Movie S7), highlights a new pathway for human‐supervised swarm teleoperations of mobile cyborgs in unknown environments, potentially utilizing multiple onboard camera agents in coordination with paramedic cyborg insects.

**FIGURE 7:**
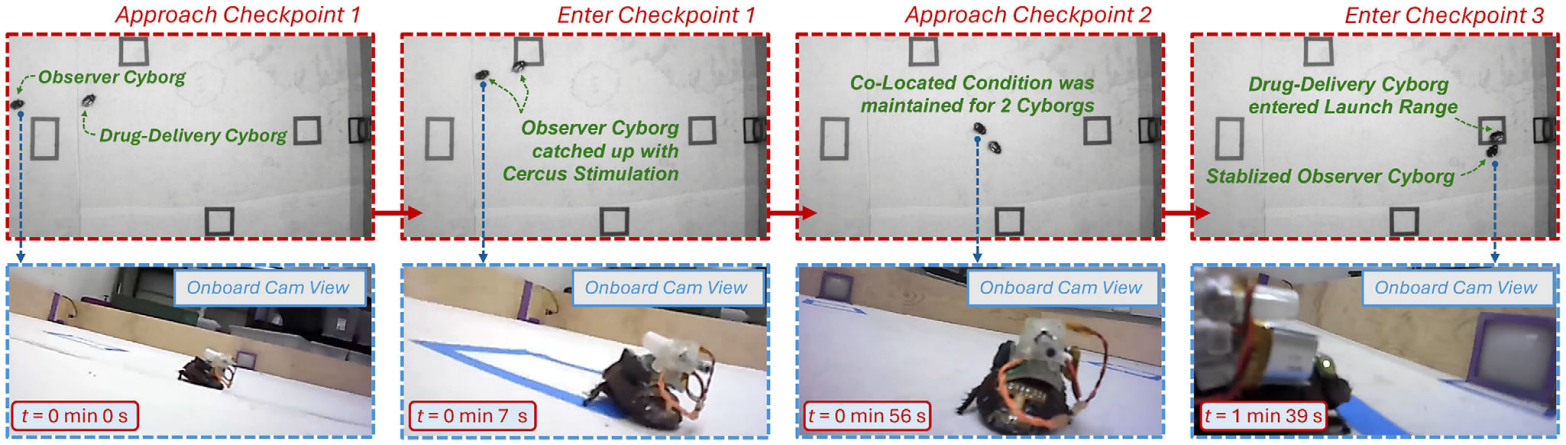
Teamwork concept for in situ drug‐delivery using two co‐located teleoperated paramedic cyborg cockroaches. Similar to the dispersed scenario, drug‐delivery is executed under real‐time supervision provided by the camera‐equipped cyborg. In this case, however, the two paramedic cyborg cockroaches maintain proximity throughout the search process, i.e., navigating via assigned checkpoints, until termination, which occurs once the onboard camera verifies that the drug‐delivery cyborg has reached the effective launch range of the AIM, i.e., passing checkpoint 3.

## 3 Discussion

### 3.1 Expanding Cyborg Insects From Search to In Situ Rescue

Building on the demonstrated reliability of the in situ drug‐delivery function performed by Paraborg with AIM, this study marks a new chapter for biohybrid mobile robots, expanding their role from solely “search” and “explore” capabilities to active “rescue” functions, such as providing first aid to trapped victims. This study presents the first comprehensive proof‐of‐concept demonstration of the paramedic cyborg insects (Paraborgs), capable of providing medical assistance in emergency scenarios, specifically, in situ drug‐delivery for trapped victims in complex terrains and confined, unknown environments. By enabling targeted in situ interventions, Paraborgs broaden the potential applications of terrestrial cyborg insects, which over the past two decades have been developed primarily for search and exploration. Substantial advances in hardware integration, autonomous control, system reliability, and field validation will be required before such platforms can be deployed in real‐world emergencies. Nevertheless, these results establish the feasibility of insect‐scale biohybrid robots for localized medical support and provide a foundation for developing robust, field‐deployable rescue systems.

### 3.2 Integrated Auto‐Injection and Locomotion Control for Drug‐Delivery

The practicality of Paraborgs for on‐field deployment alongside current emergency response teams stems from two key factors: (i) an efficient teleoperated auto‐injection mechanism (AIM), and (ii) a flexible, low‐power, self‐adaptable cyborg cockroach equipped with a reliable manual control strategy. Compared to current needle‐based injection systems, AIM stands out as one of the most effective and integrable options for small‐scale mobile robots, offering advantages such as compactness, low cost, and rapid autonomous injection with a proactive triggering mechanism. The reliability and functionality of AIM can be further enhanced through a refined fabrication procedure and additional designs, e.g., multi‐shot mechanisms. Beyond AIM, the developed manual control system also plays a critical role in the feasibility of Paraborgs, achieving a 100% success rate in reaching target checkpoints and entering the injection range. This system introduces two novel strategies compared to existing studies on cyborg insect locomotion: (i) a U‐turn counter to overcome habituation at the unilateral antenna and improve positioning near close‐range targets, and (ii) a specialized drug‐delivery control protocol combining bilateral antenna stimulation, low‐frequency unilateral stimulation, and cerci stimulation suppression to stabilize the insect during the launch process. Using both AIM and the advanced manual control system, Paraborgs achieved an overall success rate of approximately 72% for completing the full task, from locomotion through multiple checkpoints to successful in situ drug‐delivery. Notably, this success rate can increase to up to 95% when the launch is performed at a close range, within 150 mm of the target.

### 3.3 Payload Tolerance, Locomotion Robustness, and Launch Stability

Furthermore, we compared the average linear speed and maximum angular velocity of Paraborg under unloaded and maximally loaded conditions across three locomotion scenarios: (i) free walking, (ii) wall following, and (iii) corner turning. Consistent with previous cyborg‐insect studies [41, 49, 82], no significant differences were observed between the two loading conditions. These results indicate that Paraborg maintains efficient locomotion and navigation even under the heaviest payload configuration, likely because the maximum payload remains below the insect's body weight (Figures S6 and S7). The results also suggest that potential effects associated with shifts in the insect's center of gravity, increased joint torque, or additional metabolic demand were minimal under the tested conditions (Figure S6 and Movie S2). Although *Macropanesthia rhinoceros* is not inherently well suited for climbing tasks, a limitation that may be addressed in future work by selecting alternative insect platforms [83, 84], the fundamental climbing strategy was preserved under both AIM and onboard camera configurations (Figure S7 and Movie S2). In addition, experiments across five Paraborgs demonstrated that recoil generated during AIM deployment has a negligible effect under both free‐space launching and target‐contact conditions (Figure S12). Finally, the added payload from the AIM does not significantly affect overall navigation performance when sufficient recovery periods are incorporated between trials (Figure S13).

### 3.4 Translational Barriers and Safety‐Oriented Delivery Improvements

Despite the promising capabilities of Paraborg, several key limitations remain that must be addressed for real‐world deployment. The present experiments establish the feasibility of in situ drug‐delivery but were conducted under controlled conditions that do not capture the complexity of disaster environments. Post‐disaster sites may contain gravel, loose sand, uneven surfaces, collapsed debris, confined passages, and unpredictable obstacles, all of which can impair locomotion, navigation, and mission execution. Validation under progressively more realistic conditions will therefore be required to assess locomotor robustness, navigation reliability, and payload‐delivery performance. Technical challenges such as wireless communication and teleoperation via onboard camera in unstructured environments (e.g., rubble or post‐disaster settings) also present significant barriers to real‐world deployment of Paraborgs. For example, Bluetooth signals can be strongly attenuated by dense materials such as concrete and soil, whereas non‐line‐of‐sight conditions can disrupt the reliable, low‐latency transmission of high‐bandwidth video, resulting in intermittent or complete signal loss. In addition, drug–excipient compatibility and delivery at therapeutically relevant volumes remain important limitations. While an initial contamination assessment using a phenol red indicator suggests effective chemical isolation from carbon dioxide generation and citric acid byproducts at small injection volumes (0.1 mL, Figure S10), this simplified test does not fully capture potential effects on drug stability, concentration, or bioactivity. The current hardware configuration (31G × 6 mm needle and 0.5 mL reservoir) also limits the deliverable dose. Future work should therefore focus on developing more robust communication strategies, integrating multimodal sensing for resilient control and localization, and performing rigorous chemical compatibility studies (e.g., spectrophotometric or HPLC analysis), alongside system optimization to enable clinically relevant dosing.

For future deployment, unpredictable insect motion during the AIM launching process may introduce safety risks and reduce operational reliability (Table S4). To address this limitation, we propose two directions for future development: (i) close‐range launching using a low‐stiffness spring mechanism, and (ii) microneedle patch‐based delivery. In the first approach, building on the current needle‐based design, the AIM‐carrying Paraborg will be guided to establish direct contact with the target site prior to launching, thereby minimizing the likelihood of misalignment caused by sudden free‐space insect movement or an unstable standing platform. Furthermore, the compressed spring will be replaced with a lower‐stiffness alternative to reduce both launching and puncture forces, mitigating the risk of injury. In the second approach, microneedle patches present a promising pathway toward safer and more robust operation on human skin, which is multilayered, region‐dependent, curved, vascularized, deformable, and often mobile relative to the underlying tissue. The current puncture force, penetration depth, and injector stability measured on silicone targets cannot be directly interpreted as evidence of safe or effective injection into human tissue. In particular, the measured forces of approximately 6–10 N exceed the low‐newton insertion forces typically reported for controlled small‐gauge needle insertion into human skin [85, 86]. Microneedle patches, which consist of arrays of micron‐scale needles, offer a minimally invasive alternative capable of painlessly penetrating the outer skin layer while either dissolving to release therapeutic agents or creating microchannels that enable drug diffusion [87, 88]. In the proposed implementation, AIM would carry a lightweight, preloaded microneedle patch and deploy it via a simple “place‐and‐press” rather than a high‐speed “launch‐and‐inject” process. This strategy significantly reduces actuation force requirements and sensitivity to precise alignment, allowing effective delivery even in the presence of minor positional errors or small insect movements. Additionally, the use of dissolvable microneedles eliminates concerns related to needle retrieval and sharps waste, further improving overall system safety. Although these approaches demonstrate proof of principle for delivering medical treatment to trapped victims using insect‐scale search‐and‐rescue robots, including Paraborg, further development and clinical validation will be required before real‐world deployment.

### 3.5 Toward Autonomous Swarms of Specialized Paraborgs

From the successful demonstration of control strategies in both dispersed and co‐located scenarios among multiple Paraborg units, future integration of advanced swarm control algorithms, in combination with multiple onboard sensors and actuation systems, could enable the development of a fully autonomous paramedic team composed of terrestrial cyborg insects. Leveraging the concept of teamwork between multiple Paraborgs, integrating Paraborgs with compact, multimodal sensing and actuation systems, such as flexible robotic arms for in situ environmental sensing [53, 54, 55, 56, 57], multiple onboard cameras for real‐time visualization [40, 41, 42, 75], multi‐short drug‐delivery mechanisms [89, 90], and microelectronics for efficient sensing and control [91, 92, 93], its operational potential can be significantly enhanced. We vision that these developments will pave the way for a new generation of search‐and‐rescue teams composed of functionally specialized paramedic cyborg insects, capable of real‐world deployment in complex and hazardous environments. Furthermore, the demonstrated dual‐agent Paraborg framework remains vulnerable to individual‐agent failure, which could compromise the entire mission. Although this proof‐of‐concept study employs the minimum number of agents required to demonstrate autonomous in situ drug‐delivery, future rescue operations could instead deploy larger swarms. Redundancy within such swarms would allow multiple agents to independently search for victims, localize targets, relay information, and deliver medical payloads. The loss of an individual agent would therefore be less likely to prevent mission completion, improving the robustness and resilience of biohybrid rescue systems.

## 4 Experimental Section

### 4.1 Insect Platform

A giant burrowing cockroach (*Macropanesthia rhinoceros*) was employed in this study to construct a new generation of cyborgs with advanced hardware components for medical assistance, i.e., “paramedic cyborg insect” or Paraborg. This species possesses appropriate size (∼84–87 mm body length, ∼37–45 mm body width, ∼18–24 mm height, ∼34–40 g weight, up to 10 years lifespan [94], Figure 2A and Figure S2) to integrate centimetre‐scale apparatuses for further applications in real‐world scenarios. Although ethical guidelines for invertebrate research are not yet standardized [95], the cockroaches were provided with a suitable living environment (20°C and ∼86% humidity) and decent nutrition, i.e., dry gum leaves were provided biweekly while apples were fed once a week to keep the cockroaches hydrated. The nourishing colonies were cleaned biweekly before feeding the cockroaches to maintain hygiene and avoid fungus or any bacteria; humidity was maintained by adding an appropriate amount of water on the surface after the cleaning process.

### 4.2 Implantation and Control Method

Locomotion control of paramedic cyborg insects was carried out based on a similar methodology of previous terrestrial insect platforms such as Madagascar hissing cockroach (*Gromphadorhina portentosa*) [69, 71]. For navigation control, electrodes were implanted at two antennas (4–6 mm inserted depth, 127 µm diameter, Teflon‐coated bare silver wire, A‐M Systems, Figure S2). By stimulating the left antenna, the implanted cockroach performs a right turn and vice versa (Figure 2) [69, 71, 73], i.e., the higher the frequency and amplitude are, the more intense the steering response achieved from the cyborg cockroach (Figure 2B and Figure S5C) [47, 71]. For accelerating the cyborg cockroach, electrical stimulation at two cerci was employed (Figure 2B and Figure S2). Every cockroach was left to recover for at least 1 h after implantation in a small area with gum leaves and apple slices. Besides the turning and accelerating control, paramedic cyborg cockroaches were also equipped with a control strategy to perform the in situ drug‐delivery function, i.e., this strategy was initiated when the cockroach entered the launch range after reaching checkpoint 3 (Figure 4A). During this process, the cockroach was required to be stable for launching the injector. Therefore, stimulation at the cerci was prohibited while simultaneous stimulation at both antennae could be applied to immobilize the cockroach (Figure 1C). Stimulation of the antenna and both cerci requires approximately 0.4 and 2.7 µW of power, respectively (Figure S4). Using the highest power consumption associated with both cerci stimulation, the cost of transport (CoT) is estimated to be 0.23 (Section S1.3). This low CoT highlights the energy efficiency of the Paraborg and positions it among the most promising insect‐scale robotic platforms for search and rescue operations reported to date (Table S1).

### 4.3 Experiment Setup of Locomotion Control via Electrical Stimulation

Steering and accelerating behaviors of paramedic cyborg insects were studied through the response experiment in a closed arena (55 cm × 35 cm) with a 100‐fps top‐view camera for tracking and a 60‐fps side‐view camera for recording videos (Figure S11). Locomotion of the cockroach was fed to the main computer with DLC Live [96] (Figure 4A and Figure S11) to collect data of instantaneous positions, i.e., *x* and *y* coordinates of head and middle labelled points. This dataset was then used to calculate induced left/right turning angles under electrical stimulation at the antennae, and forward velocity under electrical stimulation at both cerci (Figure S1). Electrical stimulation at sensory organs was regulated through a miniature PCB (Figure S3) powered by a LiPo battery (3.7 V, 100 mAh, 30C). Monopolar pulses with fixed amplitude and duration (∼2.7 V, 500 ms, 2 ms pulse width, Figure S3B) were employed while the applied frequency was varied (10 – 20 – 30 – 40 Hz) to investigate the graded response from giant burrowing cyborg cockroaches. The experiment was carried out with 5 cockroaches; each value of frequency was applied to a cockroach with 10 electrical stimulations at the antennae (i.e., 5 at the left antenna and 5 at the right counterpart) and 5 stimulations at both cerci. In summary, each value of frequency (10–40 Hz) for a stimulated organ (left/right antennae or both cerci) consists of data from 25 stimuli. The duration between two consecutive stimuli remained ∼1–3 min.

For turning control of Paraborgs, electrical stimulation at a unilateral antenna was employed following the varying‐frequency principle from earlier studies about locomotion control of terrestrial cyborg insects [47]. Constant electrical parameters include stimulating amplitude, duration, and pulse width (∼2.7 V, 1000 ms duration, 2 ms pulse width, Figure S3), while applied frequency varied in the range 10–50 Hz to manipulate the turning magnitude of the cockroach. Several values of stimulation duration (500–1500 ms) and pulse width (1–5 ms) were investigated to determine the finest parameters for locomotion control of the giant burrowing cockroach. Considering the stimulation duration independently, although a lower value of 500 ms still induced a change in heading angle from the cockroach, the turning response occurred rapidly, and there was no considerable difference in turning angles generated by lower and higher frequencies. On the other hand, a higher duration of 1500 ms could provide significant turning angles and graded response in the frequency range 10–50 Hz; however, the cockroaches usually show a resistance behavior during turning, i.e., small inverse turns during the response duration and immobility during or after multiple stimulations, which could potentially be an indication of tissue damage and habituation [97, 98, 99]. Therefore, 1000 ms was selected for the later response and drug‐delivery experiments. For the constant pulse width, the lower value of 1 ms also shows a similar trend as the 500 ms stimulation duration, when the cockroaches performed insignificant turning responses. Under higher pulse widths of 3–5 ms, the cockroach demonstrated rapid and intense turning responses even with 10 Hz frequency while they tried to remove the implanted electrodes, which implies that the Paraborg was overloaded by the overstimulation [78]. This explains why the pulse width was eventually set to 2 ms.

Similar to previous cockroach platforms, electrical stimulation at both cerci of giant burrowing cockroaches also increases forward velocity [69, 71], which has been usually employed for acceleration control (Figure 2B). Electrical parameters for cerci stimulation also had the same amplitude as the antenna stimulation, while stimulating duration and pulse width were adjusted after initial tests (∼2.7 V, 300 ms duration, 5 ms pulse width, 10–50 Hz, Figure S3). When applying electrical stimulation at both cerci, the forward velocity was maintained stably after ∼300 ms for the frequency range 10–50 Hz even when the stimulating duration was prolonged more than this value (Figure S3C); therefore, the duration was set at 300 ms for later experiments. A constant pulse width of 5 ms was determined based on the test outcome, which shows that higher values usually damage the cerci sensory cells and produce over‐responsiveness, i.e., the abdomen section of the cockroach retracted abnormally after the initiation of electrical stimulation and response deterioration occurred rapidly.

### 4.4 Prototype Fabrication of Auto‐Injection Mechanism

The design of this auto‐injection mechanism not only shows the compact characteristic and effective capability as a first aid to increase the survival chance of victims during or after wide‐area catastrophes, but it also possesses the low‐cost feature for later commercialization. A ready‐to‐use set of the injection mechanism includes only 10 components, i.e.,, 8 mechanical parts and 2 chemical substances (Figure 3A). 8 out of 10 components are available at any suppliers of household goods or hardware stores and can be purchased with reasonable prices, i.e., compressed spring (8 mm diameter, 23 mm length), NiCr wire (0.3 mm diameter, 40 mm in length), fishing line (0.12 mm diameter, SITUATION Braided Line), injector syringe (0.5 mL, 0.25 mm (31G) × 6 mm, BD Ultra‐Fine Insulin Syringe), acid chamber (0.3 mL, 0.25 mm (31G) × 6 mm, BD Ultra‐Fine Insulin Syringe), membrane (nylon, ∼0.1 mm thickness), citric acid (C_6_H_8_O_7_, ∼60% concentration) and potassium bicarbonate powder (KHCO_3_). The prototypes of the two remaining components, i.e., launch tube and collar, were fabricated by SLA 3D printing for rapid testing and adjustment, while still ensuring cost savings and fine quality (Elegoo Mars 4 Ultra printer, Monocure 3D Rapid Model resin).

### 4.5 Experiment Setup for Navigation and Drug‐Delivery With Manual Control

To verify the performance of paramedic cyborg insects, an experimental arena was constructed (flat fabric platform, Figure S11). Every paramedic cyborg insect started its trials inside a starting region (170 mm area, Figure S11) and was then controlled manually by the remote controller to overcome a searching region (3 checkpoints, Figure S11). Across experimental trials, the average instantaneous velocity during navigation in the large arena was approximately 8.4 mm/s, as measured across five Paraborgs. Based on the total navigation distance of approximately 2500 mm between the predefined checkpoints (Figure S11C), the average time required to complete the demonstrated navigation task was estimated to be approximately 5 min. This value represents the estimated task completion time under the experimental conditions and was used as a reference for the duration of the demonstrated rescue mission rather than the maximum operational endurance of the Paraborg system.

When approaching the other edge of this searching region (i.e., checkpoint 3), the cockroach will enter a launching range (300‐mm distance to the silicone target, Figure 4A and Figure S11), where the orientation of experimental cyborg adjusted toward the injecting target, i.e., only electrical stimulation at left—right antennae with low‐frequency values 10–20 Hz were allowed while cerci stimulation was prohibited during this period. After achieving a fine orientation, the in situ drug‐delivery function was initialized to perform the auto‐injection process. Ecoflex 00–30 (durometer 00–30, 25 mm thickness) was selected as the target material due to its mechanical similarity to human skin, characterized by a low elastic modulus, high compliance, and nonlinear elastic behavior. Experimental trials of the paramedic cyborg cockroaches were recorded through a top‐view camera (∼100 fps) and tracked by another camera (∼70 fps), similar to the mentioned experiment verifying the response of paramedic cyborg cockroaches to collect the trajectory data and auto‐injection performance for post‐study (recoil analysis for postural stability of Paraborg after AIM triggering, Figure S12). The experiment was carried out with 5 cockroaches; i.e., each cockroach performed 5 trials and was left to recover for at least 30 min after every trial. Of the 25 experimental trials, 7 were performed utilizing the onboard camera. In these instances, the AIM launching and injection procedures with Paraborgs were monitored and supervised exclusively through the onboard camera feed (Wi‐Fi network, 2.4 GHz wireless, ∼24 fps, battery capacity 300 mAh, operating time 2 h), without reliance on any external tracking systems. The integration of all components for observer cyborg, including onboard camera, increased the body dimensions of the cockroach by approximately 12 mm in height and 26 g in total mass. After the experiment, implanted electrodes and plastic mounts were removed from the cockroaches; then, they were provided the same treatment as those cockroaches which have not undergone implantation in the colonies (Section S1.6).

## Author Contributions

**H. Nhan Le**, **P. Thien Phan**, **T. Nho Do**, and **T. Thang Vo‐Doan**: conceptualization. **H. Nhan Le**, and **P. Thien Phan**: data curation. **H. Nhan Le**: formal analysis. **T. Thang Vo‐Doan** and **T. Nho Do**: funding acquisition. **H. Nhan Le**, **P. Thien Phan**, **Lachlan Fitzgerald**, and **T. Thang Vo‐Doan**: investigation. **H. Nhan Le**, **P. Thien Phan**, **T. Nho Do**, and **T. Thang Vo‐Doan**: methodology. **T. Thang Vo‐Doan** and **T. Nho Do**: project administration. **T. Thang Vo‐Doan** and **T. Nho Do**: resources. **Lachlan Fitzgerald** and **H. Nhan Le**: software. **T. Thang Vo‐Doan**, **P. Ross McAree**, and **T. Nho Do**: supervision. **H. Nhan Le**, **P. Thien Phan**, **T. Nho Do**, and **T. Thang Vo‐Doan**: validation. **H. Nhan Le**, **P. Thien Phan**, **T. Nho Do**, **T. Thang Vo‐Doan**, and **Lachlan Fitzgerald**: visualization. **H. Nhan Le**, **P. Thien Phan**, **Lachlan Fitzgerald**, **T. Nho Do**, and **T. Thang Vo‐Doan**: writing – original draft. **H. Nhan Le**, **Lachlan Fitzgerald**, **P. Thien Phan**, **P. Ross McAree**, **T. Nho Do**, and **T. Thang Vo‐Doan**: writing – review & editing.

## Conflicts of Interest

The authors declare no conflicts of interest.

## Supporting information




**Supporting File 1**: advs76973‐sup‐0001‐SuppMat.pdf.

**Supporting File 2**: advs76973‐sup‐0002‐MovieS1‐S7.zip.

## Data Availability

The data that support the findings of this study are available from the corresponding author upon reasonable request.

## References

[1] “Why Are We Having So Many (Or So Few) Earthquakes?,” Has Naturally Occurring Earthquake Activity Been Increasing? (U.S. Geological Survey, 2012), https://www.usgs.gov/faqs/why‐are‐we‐having‐so‐many‐or‐so‐few‐earthquakes‐has‐naturally‐occurring‐earthquake‐activity.

[2] Natural disasters, Statista https://www.statista.com/study/10156/natural‐disasters‐statista‐dossier/.

[3] Turkey, Syria earthquake: Who is stepping up to help?, Al Jazeera, https://www.aljazeera.com/news/2023/2/6/major‐earthquake‐hits‐turkey‐syria‐which‐countries‐offered‐help.

[4] 2023 Turkey and Syria earthquake: one year on, British Red Cross, https://www.redcross.org.uk/stories/disasters‐and‐emergencies/world/turkey‐syria‐earthquake.

[5] Donors pledge €7 billion to Turkey, Syria quake recovery—DW, accessed March 20, 2023, https://www.dw.com/en/donors‐pledge‐7‐billion‐to‐turkey‐syria‐quake‐recovery/a‐65052723.

[6] G. Sumann , D. Moens , B. Brink , et al., “Multiple Trauma Management In Mountain Environments—A Scoping Review,” Scandinavian Journal of Trauma, Resuscitation and Emergency Medicine 28, no. 1 (2020): 117, 10.1186/s13049-020-00790-1. PMC7737289 33317595

[7] J. J. Boyd , G. Agazzi , D. Svajda , A. J. Morgan , S. Ferrandis , and R. L. Norris , “Venomous Snakebite in Mountainous Terrain: Prevention and Management,” Wilderness & Environmental Medicine 18, no. 3 (2007): 190–202, 10.1580/06-WEME-RA-087R.1. 17896851

[8] C. O. Quero and J. Martinez‐Carranza , “Unmanned Aerial Systems In Search And Rescue: A Global Perspective On Current Challenges And Future Applications,” International Journal of Disaster Risk Reduction 118 (2025): 105199, 10.1016/j.ijdrr.2025.105199.

[9] A. V. Nazarova and M. Zhai , “The Application of Multi‐agent Robotic Systems for Earthquake Rescue,” Robotics: Industry 4.0 Issues & New Intelligent Control Paradigms, ed. A.G. Kravets , (Springer International Publishing, 2020), 133–146, 10.1007/978-3-030-37841-7_11.

[10] R. Siegwart , M. Hutter , and P. Oettershagen , “Legged And Flying Robots For Disaster Response,” World Engineering Conference and Convention (WECC) (ETH‐Zürich, 2015), 10.3929/ethz-a-010643912.

[11] Z. Liu , W. Zhan , X. Liu , et al., “A Wireless Controlled Robotic Insect With Ultrafast Untethered Running Speeds,” Nature Communications 15, no. 1 (2024): 3815, 10.1038/s41467-024-47812-5. PMC11078929 38719823

[12] C. McFarland , A. Dhawan , R. Kumari , C. Council , M. Coad , and N. Hanson , “Field Insights for Portable Vine Robots in Urban Search and Rescue,” in 2024 IEEE International Symposium on Safety Security Rescue Robotics (SSRR) (IEEE, 2024), 190–197, 10.1109/SSRR62954.2024.10770046.

[13] K. Chen and F. Tian , “Design of Micro Search‐and‐Rescue Robot System and Analysis of Its Application in Disaster Rescue,” Highlights in Science, Engineering and Technology 76 (2023): 8–15, 10.54097/mmfhne27.

[14] H. Kabutz and K. Jayaram , “Design of CLARI: A Miniature Modular Origami Passive Shape‐Morphing Robot,” Advanced Intelligent Systems 5, no. 12 (2023): 2300181, 10.1002/aisy.202300181.

[15] P. A. der Maur , B. Djambazi , and Y. Haberthür , “RoBoa: Construction and Evaluation of a Steerable Vine Robot for Search and Rescue Applications,” in 2021 IEEE 4th International Conference on Soft Robotics (RoboSoft) (IEEE, 2021), 15–20, 10.1109/RoboSoft51838.2021.9479192.

[16] K. Jayaram , J. Shum , S. Castellanos , E. F. Helbling , and R. J. Wood , “Scaling Down An Insect‐Size Microrobot, HAMR‐VI Into HAMR‐Jr,” in 2020 IEEE International Conference on Robotics and Automation (ICRA) (IEEE, 2020), 10305–10311, 10.1109/ICRA40945.2020.9197436.

[17] X. Yang and L. Chang , “An 88‐Milligram Insect‐Scale Autonomous Crawling Robot Driven By A Catalytic Artificial Muscle,” Science Robotics 5, no. 45 (2020): aba0015, 10.1126/scirobotics.aba0015. 33022629

[18] P. E. Rybski , I. Burt , and A. Drenner , “Evaluation of the Scout Robot for Urban Search and Rescue,” in Proceedings of the AAAI Mobile Robot Competition and Exhibition Workshop, held as part of the National Conference on Artificial Intelligence (AAAI, 2001), https://aaai.org/papers/ws01‐01‐004‐evaluation‐of‐the‐scout‐robot‐for‐urban‐search‐and‐rescue/.

[19] J. Chen , D. Jin , Q. Wang , and X. Ma , “Programming Ferromagnetic Soft Materials For Miniature Soft Robots: Design, Fabrication, And Applications,” Journal of Materials Science & Technology 219 (2025): 271–287, 10.1016/j.jmst.2024.08.049.

[20] S. Tawfick and J. Pikul , “Materials Challenges For Powering Miniature Bioinspired Robots,” MRS Bulletin 49, no. 2 (2024): 100–106, 10.1557/s43577-023-00650-0.

[21] W. Haouas , M. Gauthier , and K. Rabenorosoa , “Miniaturized Soft Robotics: Recent Advances and Futures Opportunities,” Current Robotics Reports 5, no. 2 (2024): 15–27, 10.1007/s43154-024-00109-3.

[22] Z. Yang and L. Zhang , “Magnetic Actuation Systems for Miniature Robots: A Review,” Advanced Intelligent Systems 2, no. 9 (2020): 2000082, 10.1002/aisy.202000082.

[23] L. Hines , K. Petersen , G. Z. Lum , and M. Sitti , “Soft Actuators for Small‐Scale Robotics,” Advanced Materials 29, no. 13 (2017): 1603483, 10.1002/adma.201603483. 28032926

[24] M. Zhu and O. G. Schmidt , “Batteries For Small‐Scale Robotics,” MRS Bulletin 49, no. 2 (2024): 115–124, 10.1557/s43577-023-00651-z.

[25] S. Pané , P. Wendel‐Garcia , Y. Belce , X. Z. Chen , and J. Puigmartí‐Luis , “Powering And Fabrication Of Small‐Scale Robotics Systems,” Current Robotics Reports 2, no. 4 (2021): 427–440, 10.1007/s43154-021-00066-1. PMC8721937 35036926

[26] H. N. Le , L. Fitzgerald , P. T. Phan , et al., “Terrestrial Cyborg Insects for Real‐Life Applications,” Advanced Intelligent Systems 8 (2026): 202501119, 10.1002/aisy.202501119.

[27] W.‐W. VA , M. Guix , N. W. Xu , et al., “Biohybrid Robots: Recent Progress, Challenges, And Perspectives,” Bioinspiration & Biomimetics 18, no. 1 (2022): 015001, 10.1088/1748-3190/ac9c3b. 36265472

[28] C. J. Fraga , M. S. Brown , and N. W. Xu , “Advances in Invertebrate Biohybrid Robotics: Leveraging Nature for Locomotion and Sensing in Engineered Systems,” Advanced Intelligent Systems 7 (2025): 2401105, 10.1002/aisy.202401105.

[29] Y. Li and H. Sato , “Insect‐Computer Hybrid Robot,” Molecular Frontiers Journal 02, no. 01 (2018): 30–42, 10.1142/S2529732518500025.

[30] H. Siljak , P. H. J. Nardelli , and R. C. Moioli , “Cyborg Insects: Bug or a Feature?,” IEEE Access 10 (2022): 49398–49411, 10.1109/ACCESS.2022.3172980.

[31] W.‐W. VA , O. Akkus , U. A. Gurkan , H. J. Chiel , and R. D. Quinn , “Organismal Engineering: Toward A Robotic Taxonomic Key For Devices Using Organic Materials,” Science Robotics 2 (2017): aap9281, 10.1126/scirobotics.aap9281. PMC6663099 31360812

[32] K. Kai , L. D. Long , and H. Sato , “Streamlined Shape Of Cyborg Cockroach Promotes Traversability In Confined Environments By Gap Negotiation,” arXiv preprint (2024): arXiv:2410.07558, 10.48550/arXiv.2410.07558.

[33] J. H. Dirks , E. Parle , and D. Taylor , “Fatigue Of Insect Cuticle,” Journal of Experimental Biology 216, no. 10 (2013): 1924–1927, 10.1242/jeb.083824. 23393276

[34] J. H. Dirks and D. Taylor , “Fracture Toughness Of Locust Cuticle,” Journal of Experimental Biology 215, no. 9 (2012): 1502–1508, 10.1242/jeb.068221. 22496286

[35] C. Gebehart and A. Büschges , “The Processing Of Proprioceptive Signals In Distributed Networks: Insights From Insect Motor Control,” Journal of Experimental Biology 227, no. 1 (2024): jeb246182, 10.1242/jeb.246182. 38180228

[36] J. C. Tuthill and R. I. Wilson , “Mechanosensation And Adaptive Motor Control In Insects,” Current Biology 26, no. 20 (2016): R1022–R1038, 10.1016/j.cub.2016.06.070. PMC5120761 27780045

[37] Y. Baba , A. Tsukada , and C. M. Comer , “Collision Avoidance By Running Insects: Antennal Guidance In Cockroaches,” Journal of Experimental Biology 213, no. 13 (2010): 2294–2302, 10.1242/jeb.036996. 20543128

[38] C. M. Harley , B. A. English , and R. E. Ritzmann , “Characterization Of Obstacle Negotiation Behaviors In The Cockroach, Blaberus Discoidalis,” Journal of Experimental Biology 212, no. 10 (2009): 1463–1476, 10.1242/jeb.028381. 19411540

[39] M. H. Dickinson , C. T. Farley , R. J. Full , M. A. R. Koehl , R. Kram , and S. Lehman , “How Animals Move: An Integrative View,” Science 288, no. 5463 (2000): 100–106, 10.1126/science.288.5463.100. 10753108

[40] R. Li , Q. Lin , T.‐N. PT , D. L. Le , and H. Sato , “Smart Insect‐Computer Hybrid Robots Empowered With Enhanced Obstacle Avoidance Capabilities Using Onboard Monocular Camera,” npj Robotics 2, no. 1 (2024): 1–10, 10.1038/s44182-024-00010-3.

[41] T.‐N. PT , D. L. Le , B. S. Chong , et al., “Intelligent Insect–Computer Hybrid Robot: Installing Innate Obstacle Negotiation and Onboard Human Detection Onto Cyborg Insect,” Advanced Intelligent Systems 5, no. 5 (2023): 2200319, 10.1002/aisy.202200319.

[42] S. Rasakatla , T. Suzuki , W. Tenma , I. Mizuuchi , and B. Indurkhya , “Cameraroach: Various Electronic Backs Packs For Search And Rescue,” in 2021 IEEE international conference on robotics and biomimetics (ROBIO) (IEEE, 2021), 1300–1303, 10.1109/ROBIO54168.2021.9739264.

[43] Y. Bai , P. T. Tran Ngoc , H. D. Nguyen , et al., “Swarm Navigation Of Cyborg‐Insects In Unknown Obstructed Soft Terrain,” Nature Communications 16, no. 1 (2025): 221, 10.1038/s41467-024-55197-8. PMC11704230 39762294

[44] X. Huang , T. Li , and B. Yang , “A Wireless Image And Sound Sensing Cyborg Locust Based On Electrical Stimulation,” in Proceedings of the 2024 International Conference on Innovation in Artificial Intelligence (Association for Computing Machinery, 2024), 148–153, 10.1145/3655497.3655527.

[45] T. Latif , E. Whitmire , T. Novak , and A. Bozkurt , “Sound Localization Sensors for Search and Rescue Biobots,” IEEE Sensors Journal 16, no. 10 (2016): 3444–3453, 10.1109/JSEN.2015.2477443.

[46] M. Ariyanto , C. M. M. Refat , K. Yamamoto , and K. Morishima , “Feedback Control Of Automatic Navigation For Cyborg Cockroach Without External Motion Capture System,” Heliyon 10, no. 5 (2024): e26987, 10.1016/j.heliyon.2024.e26987. PMC10915385 38449606

[47] H. N. Le , H. D. Nguyen , L. Fitzgerald , P. R. McAree , H. Sato , and T. T. Vo‐Doan , “Burst Stimulation for Sustained Locomotion Control and Autonomous Navigation of Terrestrial Cyborg Beetles,” Cyborg and Bionic Systems 7 (2026): 0537, 10.34133/cbsystems.0537. PMC12968394 41808673

[48] S. Ma , Y. Chen , S. Yang , et al., “The Autonomous Pipeline Navigation of a Cockroach Bio‐Robot With Enhanced Walking Stimuli,” Cyborg and Bionic Systems 4 (2023): 0067, 10.34133/cbsystems.0067. PMC10631459 38026542

[49] M. J. Montagut Marques , Q. Yuxuan , H. Sato , and S. Umezu , “Cyborg Insect Repeatable Self‐Righting Locomotion Assistance Using Bio‐Inspired 3D Printed Artificial Limb,” npj Robotics 2 (2024): 3, 10.1038/s44182-024-00009-w.

[50] Y. Kakei , S. Katayama , S. Lee , et al., “Integration Of Body‐Mounted Ultrasoft Organic Solar Cell On Cyborg Insects With Intact Mobility,” npj Flexible Electronics 6 (2022): 78, 10.1038/s41528-022-00207-2.

[51] K. Shoji , Y. Akiyama , M. Suzuki , N. Nakamura , H. Ohno , and K. Morishima , “Biofuel Cell Backpacked Insect And Its Application To Wireless Sensing,” Biosensors and Bioelectronics 78 (2016): 390–395, 10.1016/j.bios.2015.11.077. 26655178

[52] D. V. Voronin , A. A. Abalymov , Y. I. Svenskaya , and M. V. Lomova , “Key Points in Remote‐Controlled Drug Delivery: From the Carrier Design to Clinical Trials,” International Journal of Molecular Sciences 22, no. 17 (2021): 9149, 10.3390/ijms22179149. PMC8430748 34502059

[53] K. Zhu , C. C. Nguyen , B. Sharma , et al., “Development of a Bioinspired Soft Robotic System for Teleoperated Endoscopic Surgery,” Cyborg and Bionic Systems 6 (2025): 0289, 10.34133/cbsystems.0289. PMC12159415 40510660

[54] J. E. Bernth , G. Zhang , D. Malas , G. Abrahams , B. Hayee , and H. Liu , “MORPHGI: A Self‐Propelling Soft Robotic Endoscope Through Morphing Shape,” Soft Robotics 11 (2024): 670–683, 10.1089/soro.2023.0096. 38484296

[55] M. T. Thai , P. T. Phan , H. A. Tran , et al., “Advanced Soft Robotic System for In Situ 3D Bioprinting and Endoscopic Surgery,” Advanced Science 10, no. 12 (2023): 2205656, 10.1002/advs.202205656. PMC10131836 36808494

[56] Y. Li , J. Peine , M. Mencattelli , J. Wang , J. Ha , and P. E. Dupont , “A Soft Robotic Balloon Endoscope For Airway Procedures,” Soft Robotics 9 (2022): 1014–1029, 10.1089/soro.2020.0161. PMC9595649 34813373

[57] M. Chauhan , J. H. Chandler , A. Jha , V. Subramaniam , K. L. Obstein , and P. Valdastri , “An Origami‐Based Soft Robotic Actuator For Upper Gastrointestinal Endoscopic Applications,” Frontiers in Robotics and AI 8 (2021): 664720, 10.3389/frobt.2021.664720. PMC8141740 34041275

[58] G. Arrick , D. Sticker , A. Ghazal , et al., “Cephalopod‐Inspired Jetting Devices For Gastrointestinal Drug Delivery,” Nature 636, no. 8042 (2024): 481–487, 10.1038/s41586-024-08202-5. PMC11634773 39567682

[59] A. Abramson , M. R. Frederiksen , A. Vegge , et al., “Oral Delivery Of Systemic Monoclonal Antibodies, Peptides And Small Molecules Using Gastric Auto‐Injectors,” Nature Biotechnology 40, no. 1 (2022): 103–109, 10.1038/s41587-021-01024-0. PMC8766875 34462588

[60] A. K. Dhalla , Z. Al‐Shamsie , S. Beraki , et al., “A Robotic Pill For Oral Delivery Of Biotherapeutics: Safety, Tolerability, And Performance In Healthy Subjects,” Drug Delivery and Translational Research 12, no. 1 (2022): 294–305, 10.1007/s13346-021-00938-1. PMC8677648 33604838

[61] A. Abramson , E. Caffarel‐Salvador , M. Khang , et al., “An Ingestible Self‐Orienting System For Oral Delivery Of Macromolecules,” Science 363, no. 6427 (2019): 611–615, 10.1126/science.aau2277. PMC6430586 30733413

[62] A. Abramson , E. Caffarel‐Salvador , V. Soares , et al., “A Luminal Unfolding Microneedle Injector For Oral Delivery Of Macromolecules,” Nature Medicine 25, no. 10 (2019): 1512–1518, 10.1038/s41591-019-0598-9. PMC7218658 31591601

[63] K. Aran , M. Chooljian , J. Paredes , et al., “An Oral Microjet Vaccination System Elicits Antibody Production In Rabbits,” Science Translational Medicine 9, no. 380 (2017): aaf6413, 10.1126/scitranslmed.aaf6413. 28275153

[64] F. Munoz , G. Alici , and W. Li , “A Review Of Drug Delivery Systems For Capsule Endoscopy,” Advanced Drug Delivery Reviews 71 (2014): 77–85, 10.1016/j.addr.2013.12.007. 24384373

[65] M. Yindee , P. Khumngoen , W. Manatchaiworakul , and T. Wongtawan , “Modifying A Mini Drone For Remote Drug Delivery For Wildlife Medicine,” Drone Systems and Applications 12 (2024): 1–6, 10.1139/dsa-2024-0015.

[66] J. LaRocco , Q. Tahmina , and J. Simonis , “Evaluating A Controlled Electromagnetic Launcher For Safe Remote Drug Delivery,” Technologies 12 (2024): 69, 10.3390/technologies12050069.

[67] Y. Zhang , D. Han , Z. Dou , et al., “The Interface Motion and Hydrodynamic Shear of the Liquid Slosh in Syringes,” Pharmaceutical Research 38, no. 2 (2021): 257–275, 10.1007/s11095-021-02992-3. 33619639

[68] M. R. L. Cattet , A. Bourque , B. T. Elkin , K. D. Powley , and D. B. Dahlstrom , “Evaluation of the Potential for Injury With Remote Drug‐Delivery Systems,” Wildlife Society Bulletin 34, no. 3 (2006): 741–749, 10.2193/0091-7648(2006)34[741:EOTPFI]2.0.CO;2.

[69] Z. Liu , Y. Gu , L. Yu , et al., “Locomotion Control of Cyborg Insects by Charge‐Balanced Biphasic Electrical Stimulation,” Cyborg and Bionic Systems 5 (2024): 0134, 10.34133/cbsystems.0134. PMC11223913 38975251

[70] T. T. Vo Doan , M. Y. W. Tan , X. H. Bui , and H. Sato , “An Ultralightweight And Living Legged Robot,” Soft Robotics 5, no. 1 (2018): 17–23, 10.1089/soro.2017.0038. 29412086

[71] J. C. Erickson , M. Herrera , M. Bustamante , A. Shingiro , and T. Bowen , “Effective Stimulus Parameters for Directed Locomotion in Madagascar Hissing Cockroach Biobot,” PLoS ONE 10, no. 8 (2015): 0134348, 10.1371/journal.pone.0134348. PMC4550421 26308337

[72] T. Latif and A. Bozkurt , “Line Following Terrestrial Insect Biobots,” in 2012 Annual International Conference of the IEEE Engineering in Medicine and Biology Society (IEEE, 2012), 972–975, 10.1109/EMBC.2012.6346095. 23366056

[73] R. Holzer and I. Shimoyama , “Locomotion Control Of A Bio‐Robotic System Via Electric Stimulation,” in Proceedings of the 1997 IEEE/RSJ International Conference on Intelligent Robot and Systems. Innovative Robotics for Real‐World Applications IROS'97 (IEEE, 1997), 1514–1519, 10.1109/IROS.1997.656559.

[74] H. D. Nguyen , P. Tan , H. Sato , and T. T. V. Doan , “Ultra‐Lightweight Cyborg Insect: Sideways Walking Of Remote‐Controlled Living Beetle With A Miniature Backpack,” in 2019 IEEE International Conference on Cyborg and Bionic Systems (CBS) (IEEE, 2019), 11–16, 10.1109/CBS46900.2019.9114394.

[75] V. Iyer , A. Najafi , J. James , S. Fuller , and S. Gollakota , “Wireless Steerable Vision For Live Insects And Insect‐Scale Robots,” Science Robotics 5, no. 44 (2020): abb0839, 10.1126/scirobotics.abb0839. 33022605

[76] Y. Aloimonos and C. Fermüller , “A Bug's‐Eye View,” Science Robotics 5, no. 44 (2020): abd0496, 10.1126/scirobotics.abd0496. 33022608

[77] K. Yamamoto , M. Ariyanto , C. M. M. Refat , and K. Morishima , “Multi‐Cyborg Insect‐Linked Formation for Object Transportation,” in 2023 IEEE International Conference on Mechatronics and Automation (ICMA) (IEEE, 2023), 588–593, 10.1109/ICMA57826.2023.10215744.

[78] M. Zhukovskaya , E. Novikova , P. Saari , and R. V. Frolov , “Behavioral Responses To Visual Overstimulation In The Cockroach Periplaneta Americana L,” Journal of Comparative Physiology A 203, no. 12 (2017): 1007–1015, 10.1007/s00359-017-1210-8. 28884199

[79] V. S. Gorelkin , I. Y. Severina , I. L. Isavnina , and V. L. Svidersky , “Effect Of Static Load On Motor Behavior Of The Cockroach Periplaneta Americana,” Journal of Evolutionary Biochemistry and Physiology 44, no. 3 (2008): 288–293, 10.1134/S0022093008030046. 18727411

[80] J. P. Martin , P. Guo , L. Mu , C. M. Harley , and R. E. Ritzmann , “Central‐Complex Control of Movement in the Freely Walking Cockroach,” Current Biology 25, no. 21 (2015): 2795–2803, 10.1016/j.cub.2015.09.044. 26592340

[81] T.‐N. PT , H. D. Nguyen , D. L. Le , R. Li , B. S. Chong , and H. Sato , “Gait‐Adaptive IMU‐Enhanced Exploration Strategy For Autonomous Search And Rescue With Insect‐Machine Hybrid System,” npj Robotics 3, no. 1 (2025): 20, 10.1038/s44182-025-00037-0.

[82] M. Ariyanto , X. Zheng , R. Tanaka , et al., “Biohybrid Behavior‐Based Navigation With Obstacle Avoidance For Cyborg Insect In Complex Environment,” Soft Robotics 12 (2025): 498–516, 10.1089/soro.2024.0082. 39930959

[83] L. Fitzgerald , H. N. Le , C. Crowe , and T. T. Vo‐Doan , “Inverted Climbing Control in Cyborg Beetles,” in 2025 IEEE International Conference on Cyborg and Bionic Systems (CBS) (IEEE, 2025), 318–322, 10.1109/CBS65871.2025.11267624.

[84] L. Fitzgerald , H. N. Le , R. S. Wilson , H. D. Nguyen , T. N. Do , and T. T. Vo‐Doan , “Zoborg: On‐Demand Climbing Control for Cyborg Beetles,” Advanced Science 12, no. 31 (2025): 02095, 10.1002/advs.202502095. PMC12376601 40472252

[85] L. H. Poniatowski , S. S. Somani , D. Veneziano , S. McAdams , and R. M. Sweet , “Characterizing and Simulating Needle Insertion Forces for Percutaneous Renal Access,” Journal of Endourology 30, no. 10 (2016): 1049–1055, 10.1089/end.2016.0342. 27519947

[86] M. E. Maloney , C. T. Potter , B. D. Chun , R. D. Montilla , and C. F. Schanbacher , “Comparative Analysis of Taper Point and Reverse Cutting Needles on Skin Puncture Force,” Dermatologic Surgery 51, no. 2 (2025): 144–147, 10.1097/DSS.0000000000004401. 39268943

[87] Y. C. Kim , J. H. Park , and M. R. Prausnitz , “Microneedles For Drug And Vaccine Delivery,” Advanced Drug Delivery Reviews 64 (2012): 1547–1568, 10.1016/j.addr.2012.04.005. PMC3419303 22575858

[88] J. Yu , J. Wang , Y. Zhang , et al., “Glucose‐Responsive Insulin Patch For The Regulation Of Blood Glucose In Mice And Minipigs,” Nature Biomedical Engineering 4, no. 5 (2020): 499–506, 10.1038/s41551-019-0508-y. PMC7231631 32015407

[89] J. W. McKeage , A. Z. H. Tan , and A. J. Taberner , “Large Volume Subcutaneous Delivery Using Multi‐Orifice Jet Injection,” International Journal of Pharmaceutics 649 (2024): 123605, 10.1016/j.ijpharm.2023.123605. 37981248

[90] J. W. McKeage , A. Z. H. Tan , and A. J. Taberner , “Jet Injection Through Microneedles For Large Volume Subcutaneous Delivery,” International Journal of Pharmaceutics 667 (2024): 124887, 10.1016/j.ijpharm.2024.124887. 39471887

[91] T. B. Dang , T. A. Truong , C. C. Nguyen , et al., “Flexible, Wearable Mechano‐Acoustic Sensors For Body Sound Monitoring Applications,” Nanoscale 17, no. 16 (2025): 9652–9685, 10.1039/D4NR05145A. 40145538

[92] J. Davies , M. T. Thai , T. T. Hoang , et al., “A Stretchable Filament Sensor With Tunable Sensitivity for Wearable Robotics and Healthcare Applications,” Advanced Materials Technologies 8, no. 6 (2023): 2201453, 10.1002/admt.202201453.

[93] Y. Qiu , A. Ashok , C. C. Nguyen , Y. Yamauchi , and T. N. Do , “Integrated Sensors for Soft Medical Robotics,” Small 20, no. 22 (2024): 2308805, 10.1002/smll.202308805. 38185733

[94] L. Xu , E. P. Snelling , and R. S. Seymour , “Burrowing Energetics Of The Giant Burrowing Cockroach Macropanesthia Rhinoceros: An Allometric Study,” Journal of Insect Physiology 70 (2014): 81–87, 10.1016/j.jinsphys.2014.09.005. 25257537

[95] J. Dabiri , O. Lenczewska , S. Wieten , C. Federico , and J. Dabiri . Ethics Of Biohybrid Robotic Jellyfish Modification And Invertebrate Research Preprints, accessed October 01 2020, https://www.preprints.org/manuscript/202010.0008/v1.

[96] G. A. Kane , G. Lopes , J. L. Saunders , A. Mathis , and M. W. Mathis , “Real‐Time, Low‐Latency Closed‐Loop Feedback Using Markerless Posture Tracking,” eLife 9 (2020): 61909, 10.7554/eLife.61909. PMC7781595 33289631

[97] R. F. Thompson , “Habituation: A History,” Neurobiology of Learning and Memory 92, no. 2 (2009): 127–134, 10.1016/j.nlm.2008.07.011. PMC2714193 18703156

[98] C. H. Rankin , T. Abrams , R. J. Barry , et al., “Habituation Revisited: An Updated And Revised Description Of The Behavioral Characteristics Of Habituation,” Neurobiology of Learning and Memory 92, no. 2 (2009): 135–138, 10.1016/j.nlm.2008.09.012. PMC2754195 18854219

[99] S. S. Haupt and W. Klemt , “Habituation And Dishabituation Of Exploratory And Appetitive Responses In The Honey Bee ( L.),” Behavioural Brain Research 165, no. 1 (2005): 12–17, 10.1016/j.bbr.2005.06.030. 16143409

