# Supplementary material for: Paraborg: Paramedic Cyborg Insects for In Situ Emergency Drug‐Delivery
Source: Adv Sci (Weinh). 2026 Aug 5;13(53):e76973. doi: 10.1002/advs.76973 (PMC13440206; doi:10.1002/advs.76973)
Supplement: Supplementary file 1 [file ADVS-13-e76973-s002.pdf]

Supporting Information

Title: Paraborg: Paramedic Cyborg Insects for In-Situ Emergency Drug Delivery

H. Nhan Le, P. Thien Phan, Lachlan Fitzgerald, P. Ross McAree, T. Nho Do and T. Thang Vo-Doan**

1. Supplementary Material for the Cyborg *Macropanesthia rhinoceros*

1.1. Data Analysis

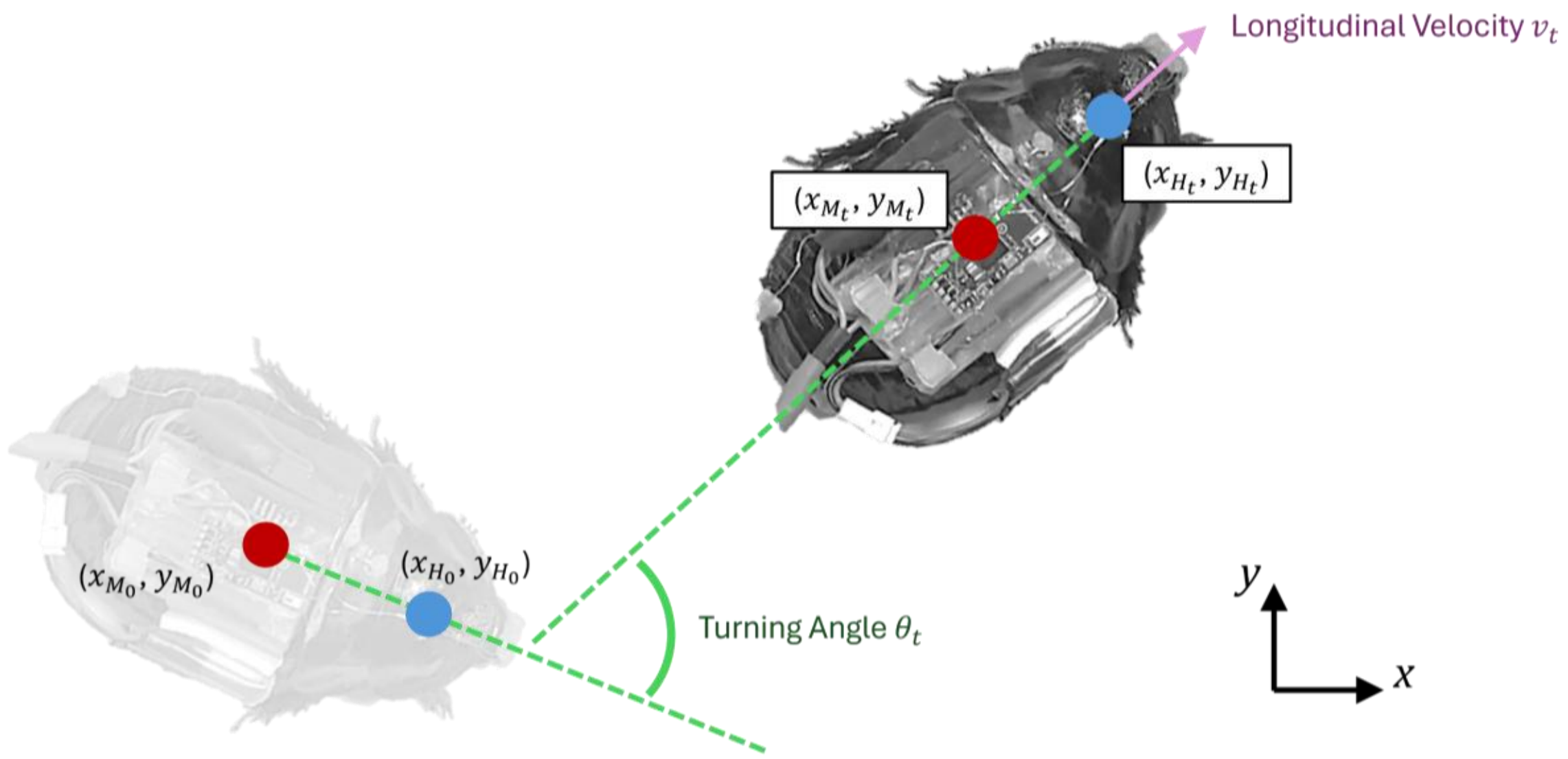

Figure S1. Overview of the calculations used in the post-experiment analysis. For the turning response induced by electrical stimulation at the antenna, the behavioral metric is defined as the turning angle over time, calculated as the angular deviation between the cockroach's orientation at time t $(x_{H_t}, y_{H_t}, x_{M_t}, y_{M_t})$ and its orientation at the onset of stimulation $(x_{H_0}, y_{H_0}, x_{M_0}, y_{M_0})$. For the accelerating response produced by electrical stimulation of both cerci, the primary metric considered is the longitudinal velocity along the body axis of the cockroach.

Turning response:

$$\theta_t = \left| \tan^{-1}\left[\frac{y_{H_t} - y_{M_t}}{x_{H_t} - x_{M_t}}\right] - \tan^{-1}\left[\frac{y_{H_0} - y_{M_0}}{x_{H_0} - x_{M_0}}\right] \right| \quad (1)$$

Accelerating response, a time step of dt = 0.01 s was used, consistent with the ~100 fps recording rate:

$$v_t = \cos\left[\theta_t - \tan^{-1}\left(\frac{y_{H_t} - y_{M_t}}{x_{H_t} - x_{M_t}}\right)\right] \sqrt{\left(\frac{x_t - x_{t-\mathrm{d}t}}{\mathrm{d}t}\right)^2 + \left(\frac{y_t - y_{t-\mathrm{d}t}}{\mathrm{d}t}\right)^2} \quad (2)$$

1.2. Experimental Supplement

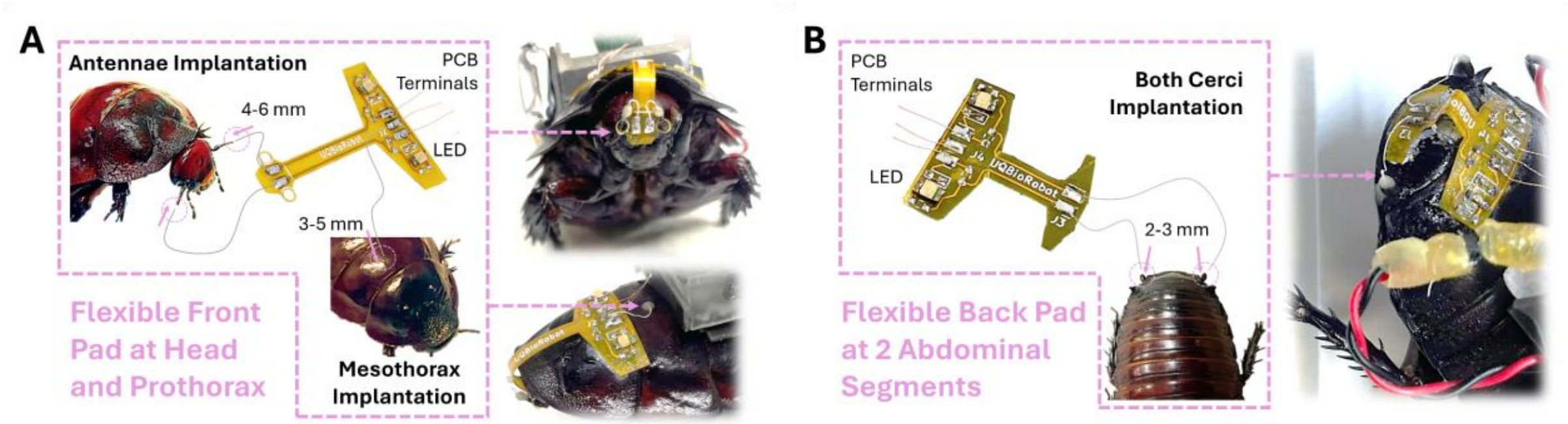

Figure S2. Integrated hardware for locomotion control of a living cockroach. (A) Front pad for turning control. A flexible pad was attached to the cockroach's head using superglue. Electrodes from the pad terminals were implanted at three positions: the two antennae (each trimmed at ~8 mm from the base, with silver electrodes inserted into the exposed slot to a depth of ~4–6 mm) and the center of the mesothorax (a hole bored to a depth of ~3–5 mm using an insect pin, #00 Indigo Instruments). Each antenna electrode was connected in parallel to an LED to indicate electrical stimulation duration. (B) Back pad for acceleration control. A silver electrode was implanted into each cercus after boring a ~3 mm deep hole with insect pin.

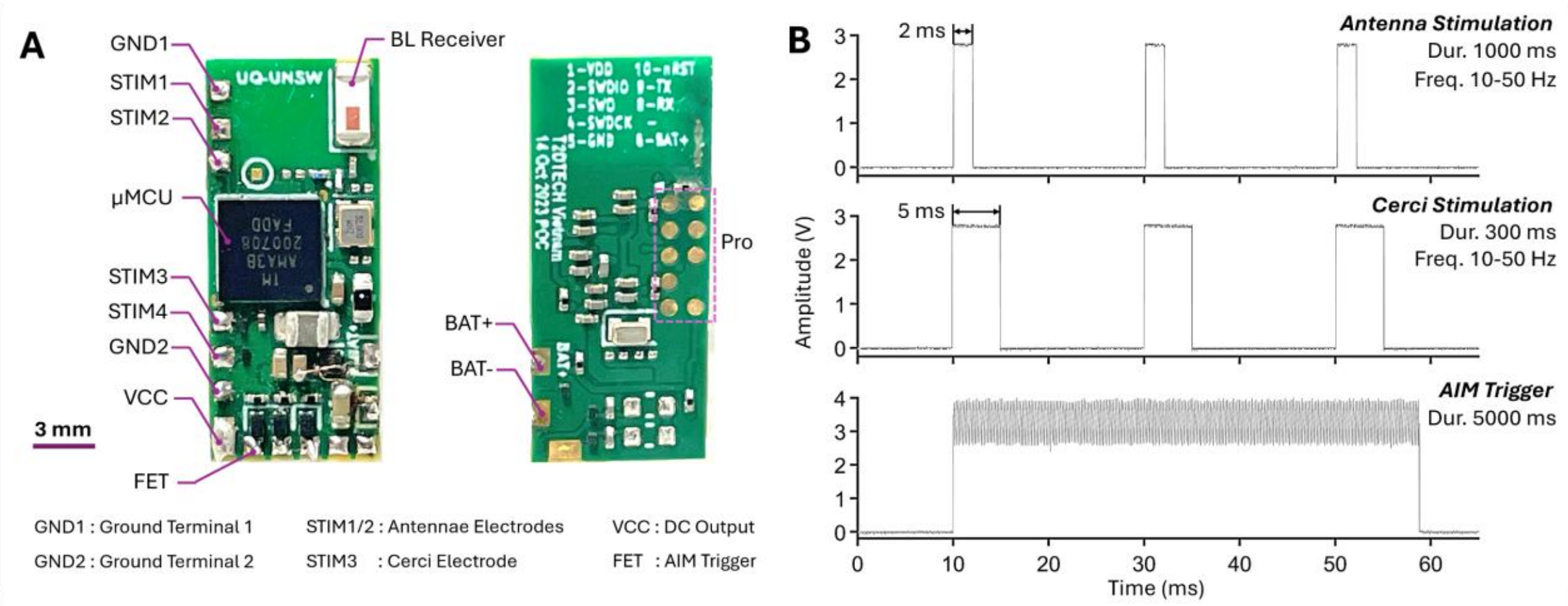

Figure S3. Overview of the integrated PCB and electrical output parameters. (A) Main components of the miniature PCB. The board includes a Bluetooth receiver for remote control, STIM terminals for stimulating implanted organs of the living cockroach, and FET terminals for triggering external components such as AIM module. A compact LiPo battery (3.7 V, 100 mAh) was used to power all integrated components during experiments. (B) Electrical outputs for locomotion control and triggering drug-delivery function of the Paraborg. For locomotion control, electrical stimuli were applied to one antenna or both cerci at ~2.7 V amplitude, with frequencies varied between 10–50 Hz. Pulse width and stimulation duration were adjusted for antenna and cerci based on preliminary response testing. To trigger AIM, a continuous signal (~3.6 V, 5 s) was applied to heat the connected NiCr wire.

1.3. Cost of Transport

To evaluate the locomotion efficiency of Paraborg, we employed the dimensionless cost of transport (*CoT*), a metric that quantifies the energy required to move a unit weight over a unit distance, as defined in Equation (3).

$$CoT = \frac{P}{mgv} \tag{3}$$

Where: P is the power consumption of the system.

m = 60 g is the average mass of a giant burrowing cockroach and onboard hardware.

g = 9.81 m/s^2 is the gravitational acceleration.

v is the linear speed of Paraborg. Higher velocity v_{free} = 34 mm/s is for free walking condition and lower velocity v_{wall} = 30 mm/s is for wall following condition (Figure S6).

For power consumption, we consider two operating scenarios: (i) Paraborg freely exploring the environment without navigation control via electrical stimulation, and (ii) Paraborg being actively guided along a desired trajectory using electrical stimulation of the antennae and both cerci. In the first scenario, Paraborg is allowed to move naturally through the environment without receiving any stimulation commands. Consequently, power consumption is only from the wireless control PCB operating in Bluetooth receiving mode, with no additional energy expenditure associated with electrical stimulation P_{PCB} = 4.07 mW, as defined in Equation (4).

$$P_{PCB} = U_{BAT}\, I_{PCB} = 3.7 \times 1.1 = 4.07 \text{ mW} \tag{4}$$

Where: U_{PCB} = 3.7 V is the nominal voltage of the LiPo battery.

I_{PCB} = 1.1 mA is the operating current measured across the PCB.

In the second scenario, when Paraborg is actively controlled with electrical stimulation, the total power consumption P_{SUM} = 4.0727 mW will include the power from the PCB P_{PCB} and the power from electrical stimulation P_{STIM}, as defined in Equation (5).

$$P_{SUM} = P_{PCB} + P_{STIM} = 4 + 0.0027 = 4.0727 \text{ mW} \tag{5}$$

Where: P_{STIM} is the power for electrical stimulation. P_{STIM} is estimated to be 0.4 µW for antenna stimulation (1 s duration) and 2.7 µW for both cerci stimulation (500 ms duration), as defined in Equation (6). Since antenna and cerci stimulation are not applied simultaneously during navigation, P_{STIM} = 2.7 µW is considered using the higher power associated with both cerci stimulation.

$$P_{STIM} = U_{STIM}\, I_{STIM} \tag{6}$$

U_{STIM} = 2.7 V is the output voltage of the stimulation channels.

I_{STIM} is the current profile when applying the stimulation to the cockroach antenna or both cerci, which was experimentally measured (Figure S4).

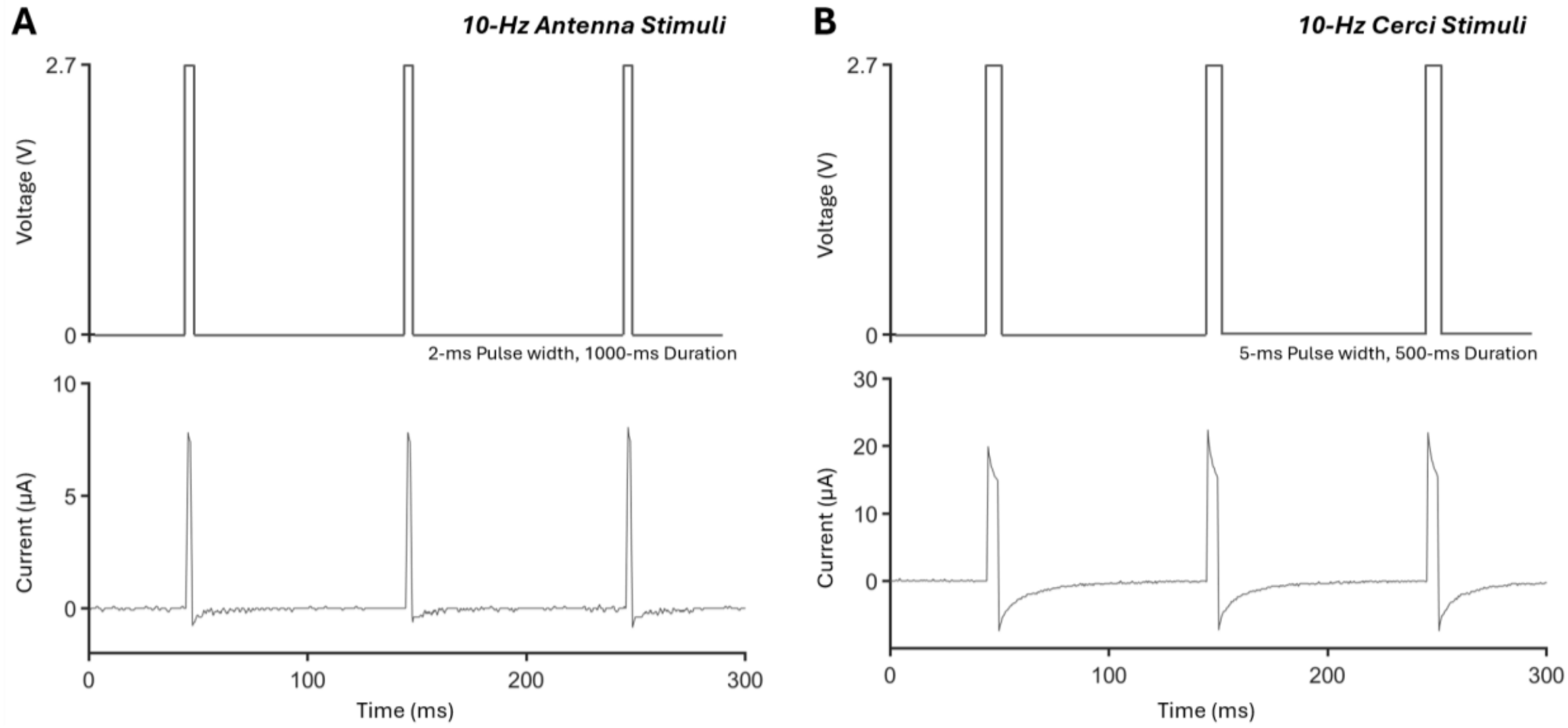

Figure S4. Parameters used for estimating the power consumption of electrical stimulation. (A) Applied voltage and corresponding electrical current during antenna stimulation. (B) Applied voltage and corresponding electrical current during both cerci stimulation. To estimate the power consumption associated with antenna and cerci stimulation, the electrical current was measured for each stimulation site.

Substitute the two values of consumption power and two average linear speeds into Equation (3), we can achieve four *CoT*s of Paraborg in different conditions:

Natural exploration in free space condition (P_{PCB}, v_{free}):

$$CoT_{PCB\_free}=\frac{P_{PCB}}{mgv_{free}}\approx 0.19$$

Natural exploration in obstructed condition (P_{PCB}, v_{wall}):

$$CoT_{PCB\_wall}=\frac{P_{PCB}}{mgv_{wall}}\approx 0.22$$

Navigation control in free space condition (P_{SUM}, v_{free}):

$$CoT_{SUM\_free}=\frac{P_{SUM}}{mgv_{free}}\approx 0.20$$

Navigation control in obstructed condition (P_{SUM}, v_{wall}):

$$CoT_{SUM\_wall}=\frac{P_{SUM}}{mgv_{wall}}\approx 0.23$$

Considering the worst-case scenario, we adopted cost of transport of 0.23 for Paraborg.

1.4. Estimation of Onboard Hardware Operational Endurance

It should be noted that these estimates represent theoretical upper bounds based solely on the power consumption of wireless communication and control electronics. During actual deployment, additional power is consumed by the electrical stimulation required for cyborg locomotion control, the frequency and duration of which depend strongly on the navigation task, environmental complexity, and individual insect responses. Consequently, the actual operating time is expected to be lower than the theoretical estimates reported below. Because stimulation usage varies substantially across missions and individuals, providing a representative estimate of stimulation-related power consumption is challenging and falls beyond the scope of the present study.

For drug-delivery Paraborg, the operational endurance is estimated by the time that Paraborg can be controlled with electrical stimulation after triggering the onboard auto-injection mechanism (AIM), T_{AIM} = 89.15 hours, as defined in Equation (7).

$$T_{AIM} = \frac{C_{re\text{-}BAT}}{I_{PCB}} = \frac{C_{BAT} - C_{AIM}}{I_{PCB}} \tag{7}$$

Where: $C_{re\text{-}BAT}$ is the remaining capacity of the LiPo battery after triggering AIM.

I_{PCB} = 1.1 mA is the measured maximum operating current across the PCB.

C_{BAT} = 100 mAh is the capacity of the LiPo battery.

C_{AIM} is the capacity needed for triggering AIM.

$$C_{AIM} = I_{AIM}\, t_{trigger} = 2000 \times \frac{3.5}{3600} = 1.94 \text{ mAh} \tag{8}$$

I_{AIM} = 2 A is the required current for triggering AIM.

$t_{trigger}$ = 3.5 s is the duration for triggering AIM.

$$T_{AIM} = \frac{100 - 1.94}{1.1} \approx 89.15 \text{ hours}$$

For observer Paraborg, the operational endurance is estimated by the time that Paraborg can be controlled by the PCB with the original capacity of the LiPo battery.

$$T_{CAM} = \frac{C_{BAT}}{I_{PCB}} = \frac{100}{1.1} \approx 90.91 \text{ hours} \tag{9}$$

However, the onboard camera is powered by a separate battery and supports wireless image transmission via Wi-Fi for a maximum of approximately 2 hours. Therefore, the operational duration of the observer Paraborg was assumed to be 2 hours, as it is constrained by the camera battery rather than the power consumption of the onboard control system.

1.5. Locomotion Performance

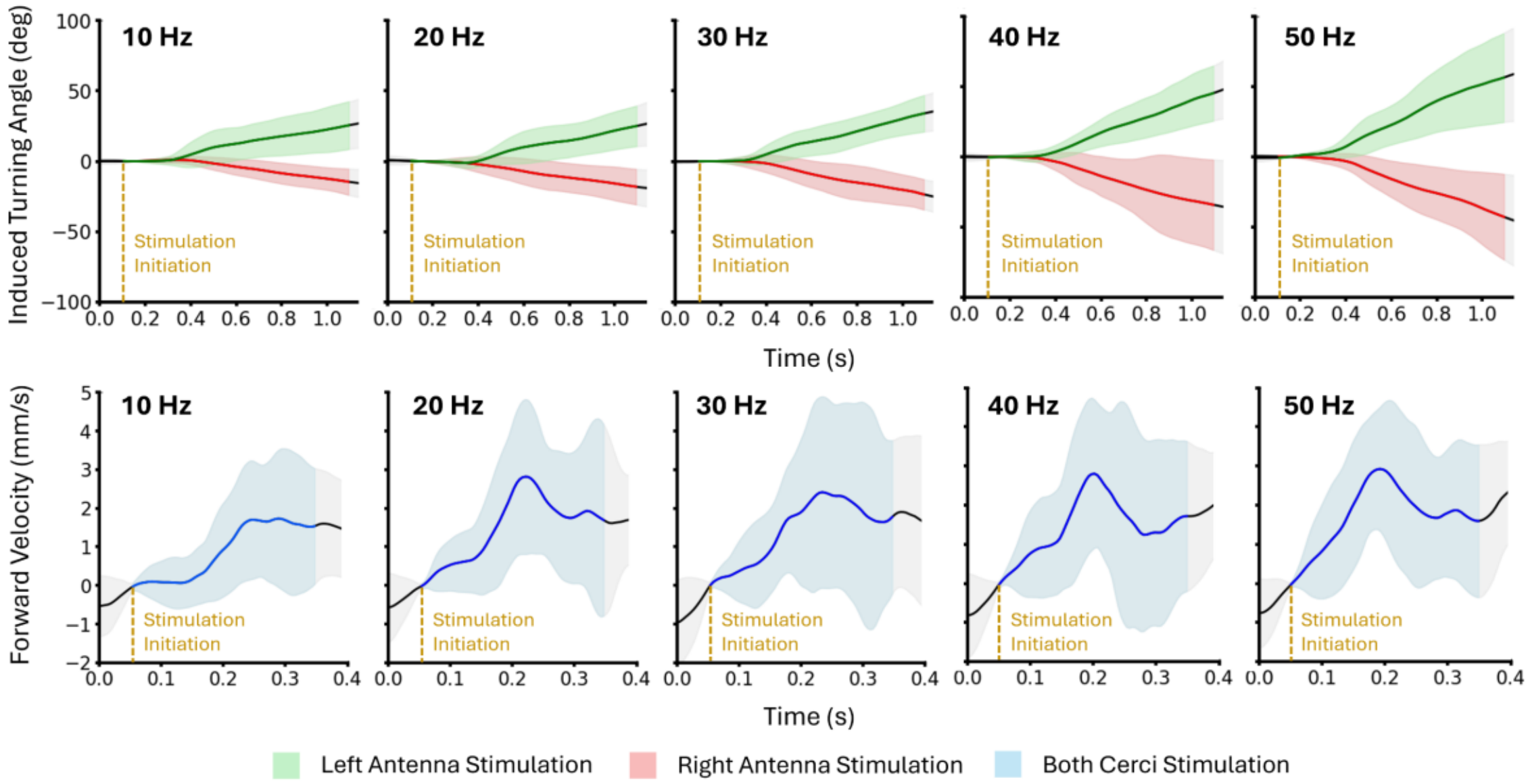

Figure S5. Motor responses of the giant burrowing cockroach under electrical stimulations at corresponding sensory organs. Data is shown as average (continuous lines) and upper/lower standard deviations (shaded region).

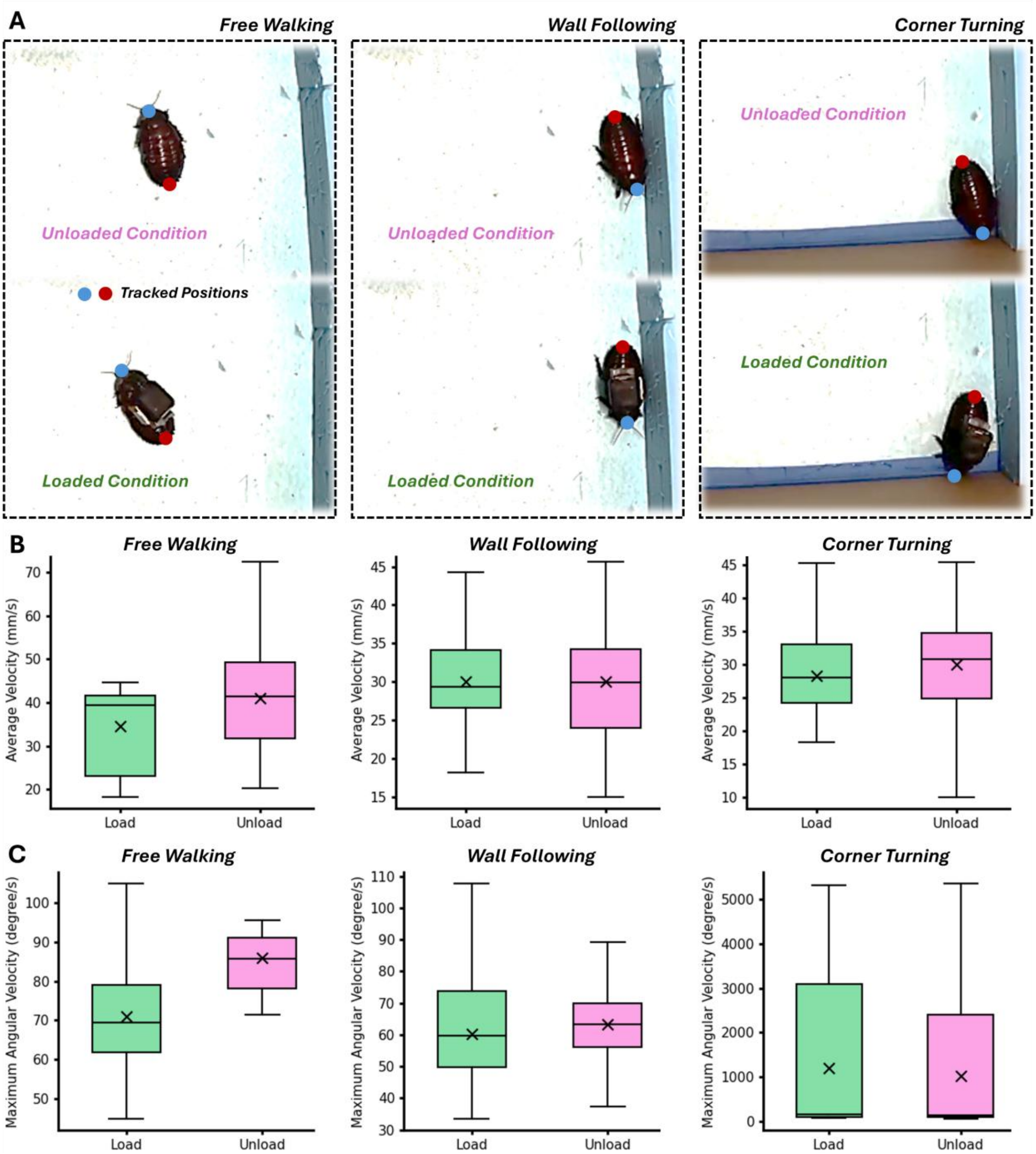

Figure S6. Investigation of locomotion performance of Paraborg under integrated hardware loading. (A) Categorized experimental conditions designed to mimic real-world locomotion scenarios. To evaluate the impact of loading on locomotion efficiency (i.e. the cockroach was integrated with the heaviest onboard camera configuration, 26 g), three experimental cases were conducted using three cockroaches: (i) free walking, representing movement in open environments; (ii) wall following, representing navigation in obstacle-rich environments; and (iii) corner turning, representing rapid maneuvering scenarios. (B) Analysis of average linear speed. Changes in locomotion performance between unloaded and loaded conditions were quantified by comparing average linear speed across multiple cockroaches (*N* = 3, *n* > 50 trials per condition for each case). (C) Analysis of angular velocity. Changes in navigational performance were assessed by comparing the maximum angular velocity per trial between unloaded and loaded conditions across multiple cockroaches (*N* = 3, *n* > 50 trials per condition for each case).

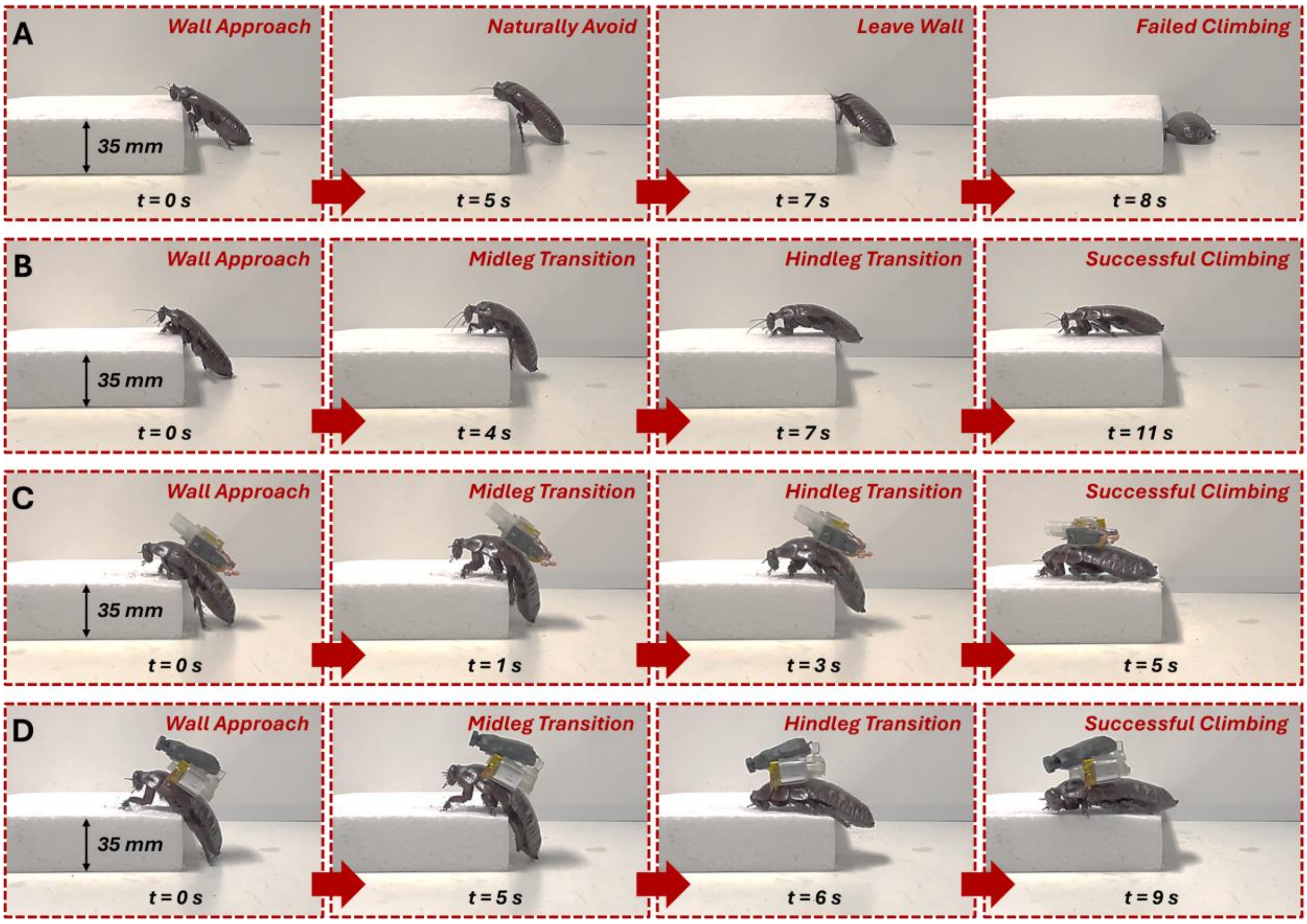

Figure S7. Investigation of climbing capability under integrated hardware loading conditions. (A) Natural obstacle-avoidance behaviour. (B) Successful climbing under the unloaded condition. (C) Successful climbing under AIM-loaded condition. (D) Successful climbing under camera-integrated condition. Across all successful climbing trials, giant burrowing cockroaches exhibited consistent climbing kinematics, transitioning onto vertical surfaces in a foreleg–midleg–hindleg sequence. The forelegs generated the primary holding and propulsive forces, enabling elevation of the body onto the higher platform.

1.6. Ethics and Post-Experiment Wellbeing Conditions

Although ethical regulations for invertebrate research remain under debate (1), we adopted a precautionary welfare approach in this study. Both intact and post-experimental giant burrowing cockroaches were maintained under clean and stable husbandry conditions, including appropriate airflow, temperature, humidity, hygiene, and weekly feeding with gum leaves and fresh apple slices. Because questions regarding insect pain perception (2,3), cognition (4), and agency remain unresolved, we treated the insects as if they could experience discomfort. Accordingly, implantation procedures were performed under anesthesia, followed by a recovery period before experimentation. After the trials, implanted electrodes were carefully removed, and the cockroaches were returned to the colony rather than euthanized or discarded.

To assess post-experimental condition, we monitored three primary indicators: (i) survival and lifespan; (ii) maintenance of normal locomotor and behavioral functions, such as wall-following behavior, escape responses to changes in lighting or gentle tactile stimuli inside the colony, regular feeding, and stable body weight; and the ability to mate and produce offspring.

All trials of five cockroaches in the drug delivery experiment were conducted between June and July 2025. As of June 2026, four of the five experimental cockroaches remain alive and appear healthy. One individual died approximately three months after being returned to the colony; however, this cockroach had already shown a gradual decline in body weight before and during the experimental period. In contrast, the four surviving individuals continue to display normal species-specific behaviors, including spontaneous exploration, wall-following locomotion, rapid escape responses, regular feeding, and body weights comparable to those of intact colony members, i.e. all experimental cockroaches have maintained body weights above 37 g. Importantly, all four survivors have successfully mated and collectively produced two generations of offspring after the experiments. Because successful reproduction depends on the maintenance of neural, metabolic, and reproductive functions, these observations suggest that the implantation and stimulation procedures did not cause obvious severe long-term physiological impairment. While transient stress or subtler physiological effects cannot be excluded, the majority of experimental cockroaches retained long-term viability, normal behavioral function, and reproductive capability after the trials.

Nevertheless, we acknowledge that the present study was not designed to systematically examine long-term physiological effects, such as neural habituation, tissue damage, or lifespan reduction, which may arise from repeated high-intensity electrical stimulation. In biohybrid robotics, understanding the long-term physiological and behavioral consequences of stimulation remain an important unresolved challenge. To the best of our knowledge, no comprehensive framework has yet been established for evaluating the post-experimental condition and welfare of insects used in cyborg or biohybrid robotic systems. Addressing this issue will require dedicated longitudinal studies involving larger sample sizes instead of only 5 individuals in this study, extended observation periods, and integrated behavioral, physiological, and histological assessments. We therefore consider the development of such evaluation methodologies, together with systematic investigation of long-term stimulation effects, an important direction for future research.

2. Supplementary Material for the Auto-Injection Mechanism

2.1. Estimation of Injection Force Generated by Chemically Produced Pressure

Balanced chemical reaction for pressure generation:

$$C_6H_8O_7+3KHCO_3 \rightarrow K_3C_6H_5O_7+3CO_2+3H_2O$$

Mass of 60% citric acid in behind chamber: $m_{C_6H_8O_7}= 0.107$ g
Mass of potassium bicarbonate in the front chamber: $m_{KHCO_3}= 0.075$ g
Air volume in the gap between the two chambers: $V_{gas}= 0.005$ litre
Absolute temperature: $T = 298.15$ K
Estimated reaction efficiency: $\eta = 85\%$

Estimation of generated pressure:

$$n_{C_6H_8O_7}=\frac{0.6\times m_{C_6H_8O_7}}{M_{C_6H_8O_7}}=\frac{0.6\times m_{C_6H_8O_7}}{192.12}\approx 3.3\times 10^{-4} \text{ mol}$$

$$n_{KHCO_3}=\frac{m_{KHCO_3}}{M_{KHCO_3}}=\frac{m_{C_6H_8O_7}}{100.12}\approx 7.5\times 10^{-4} \text{ mol}$$

$$=> n_{CO_2}=\eta\times\min\left(3n_{C_6H_8O_7}, n_{KHCO_3}\right)\approx 6.4\times 10^{-4} \text{ mol} \quad (10)$$

Liquid volume, which includes initial water from 60% citric acid and reaction water:

$$V_{liq}=\frac{0.4\times m_{C_6H_8O_7}}{1000}+\frac{18\times n_{CO_2}}{1000}= 5.4\times 10^{-5} \text{ litre} \quad (11)$$

Generated pressure considering dissolution:

$$P_{CO_2}=\frac{n_{CO_2}}{\frac{V_{gas}}{RT}+k_H\times V_{liq}}\approx 3.1 \text{ atm}\approx 314.1 \text{ kPa} \quad (12)$$

where $k_H \approx 0.033$ mol $L^{-1}atm^{-1}$ following Henry's law at 298.15 K and gas constant $R \approx 0.082057$ litre atm $mol^{-1}K^{-1}$.

- Estimated injection force acting on the rubber stopper:

$$F = P_{CO_2}\times A_{syringe}= 2.2 \text{ N} \quad (13)$$

where $A_{syringe}= \pi R^2_{in\_syringe}$ and $R_{in\_syringe} \approx 1.5$ mm is the inner radius of the syringe.

2.2. Estimation of Injector Launch Force from Spring Compression

Launch force from compressed spring:

$$F_{launch}= K\times u \approx 5.2 \text{ N} \quad (14)$$

where the spring constant K= 650 N/m and spring displacement due to compression $u \approx 8$ mm after finishing fabrication procedure.

2.3. Experimental Supplement

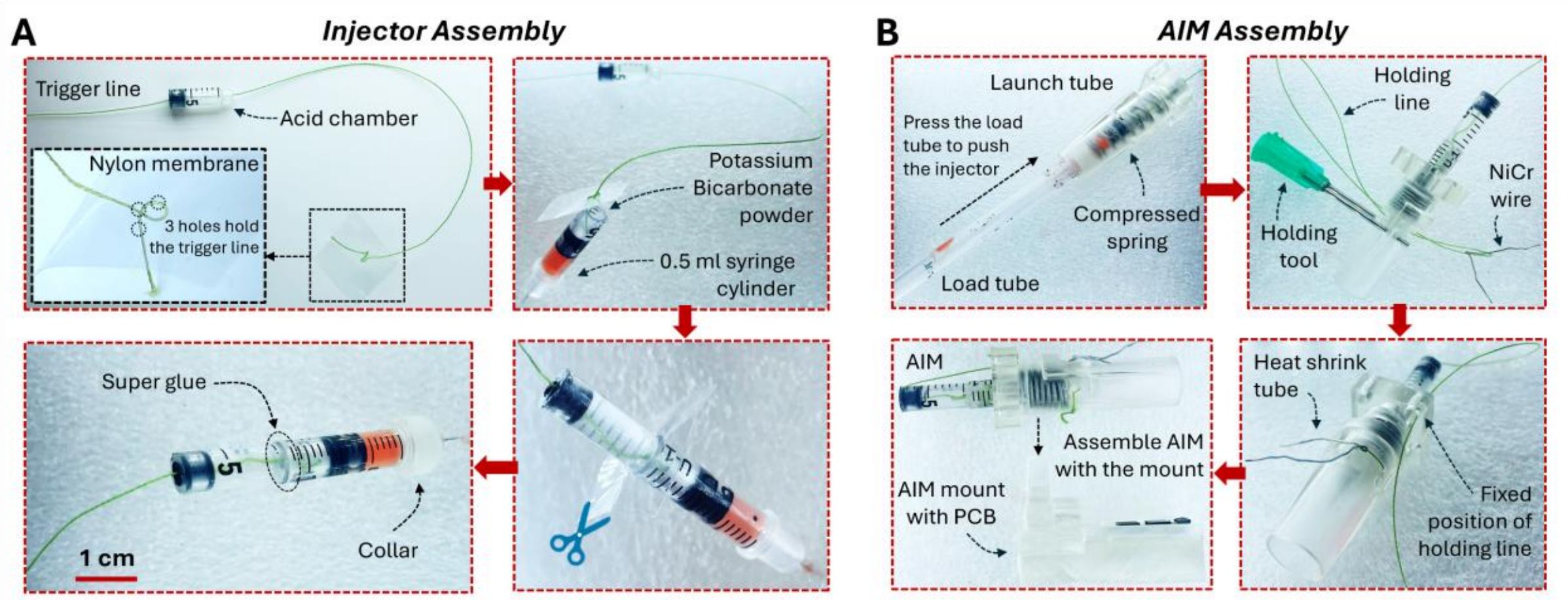

Figure S8. Fabrication procedure of the auto-injection mechanism (AIM). (A) Injector assembly process. During assembly, the injector should be held vertically with the acid chamber on top to prevent accidental leakage of potassium bicarbonate powder and unintended chemical reactions. After applying superglue to the edges of the front and rear syringe cylinders, the injector should be left to dry for approximately 10–15 minutes. (B) AIM module assembly with PCB integration. The launch tube should be replaced after every three launches to maintain its ability to securely hold the retention line while the spring is compressed. The injector can be reused multiple times in case of having no undesired chemical leakage after applying superglue.

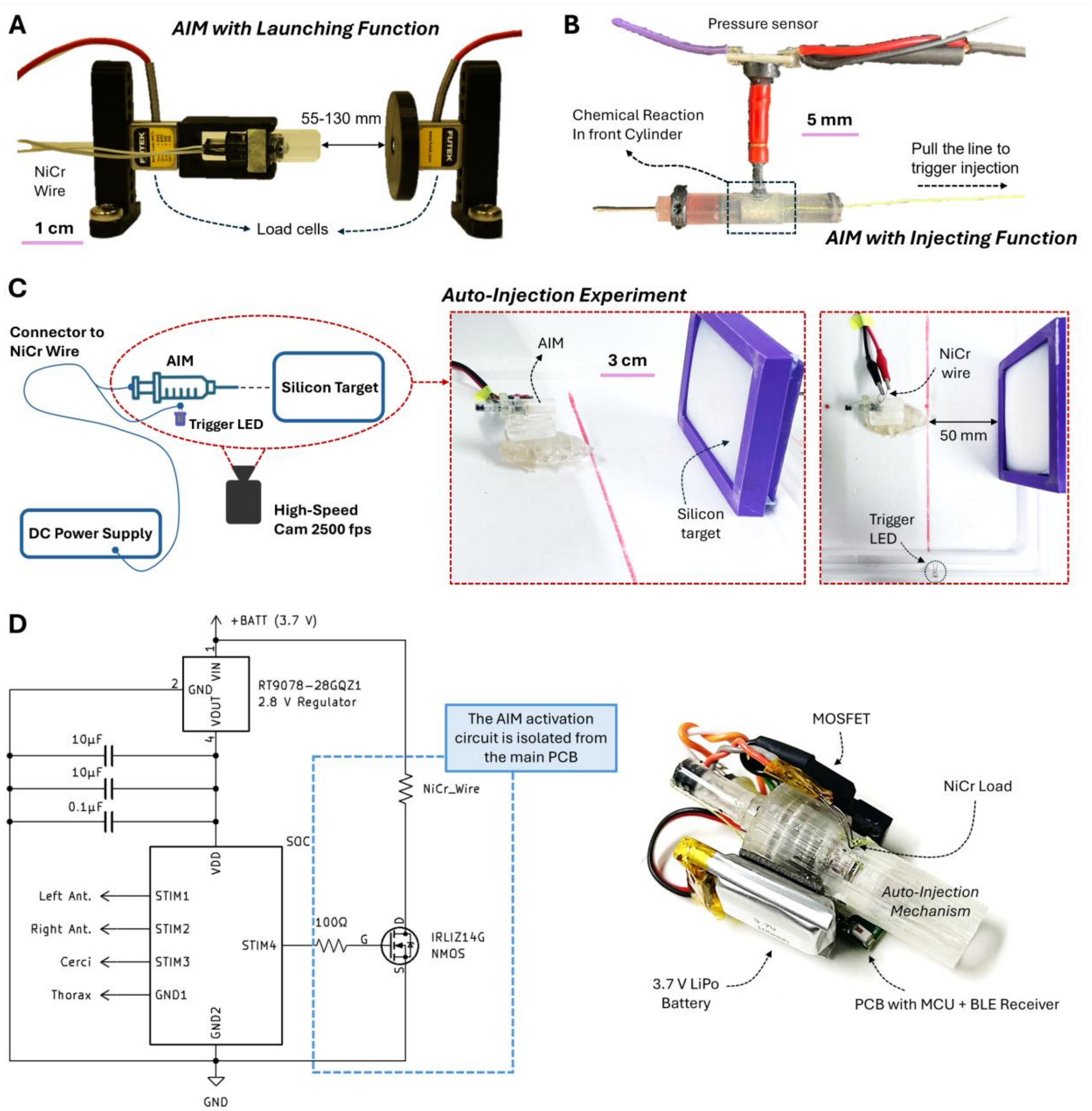

Figure S9. Experimental setup for verifying the functions of the auto-injection mechanism (AIM). (A) Launching function test. Measurement of launching and piercing forces. (B) Auto-injection test. Measurement of pressure generated in the front syringe cylinder from the chemical reaction. (C) Full drug-delivery test using a ready-to-use AIM module. The AIM was triggered by an electrical signal from a DC power supply, having similar electrical parameters to the PCB's FET output. An LED indicator was placed within the high-speed camera's view to mark the initiation of the electrical signal. (D) Electrical diagram of the onboard AIM activation system. Upon receiving the trigger command via Bluetooth communication, the MCU generates only a low-current gate-control signal to activate the MOSFET. The AIM ignition element (NiCr wire) is powered directly by the 100 mAh, 30C LiPo battery through a dedicated high-current pathway, thereby electrically isolating the control electronics from the transient ~2 A current required for AIM activation. Consequently, the high-current ignition event bypasses the MCU and wireless communication circuitry, reducing the risk of voltage sag, controller reset, or communication failure during operation.

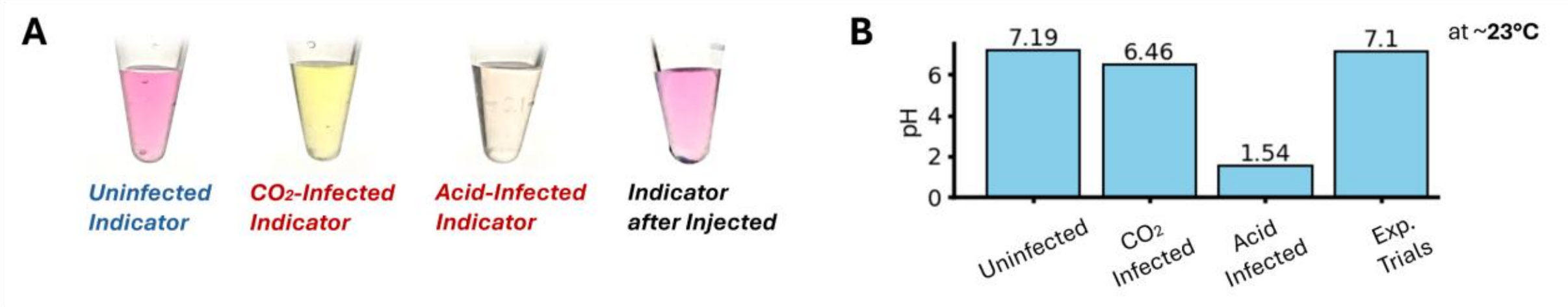

Figure S10. Drug contamination assessment during autonomous AIM injection. (A) Color-based indicator for contamination detection. The experimental setup followed that in Figure S9C, with the injected chemical (red solution) replaced by a phenol red solution (pH ≈ 7.2). This configuration enabled detection of potential contamination from: (i) carbon dioxide generated during actuation, which would shift the indicator from pink to light yellow; and (ii) citric acid from the rear chamber, which would shift the indicator from pink to transparent. The test was performed over 8 trials, with an injection volume of 0.1 mL per trial. (B) Quantitative pH measurements. Using a pH meter, the average pH across all trials was ~7.1, consistent with the unperturbed indicator solution, confirming effective chemical isolation in the AIM configuration.

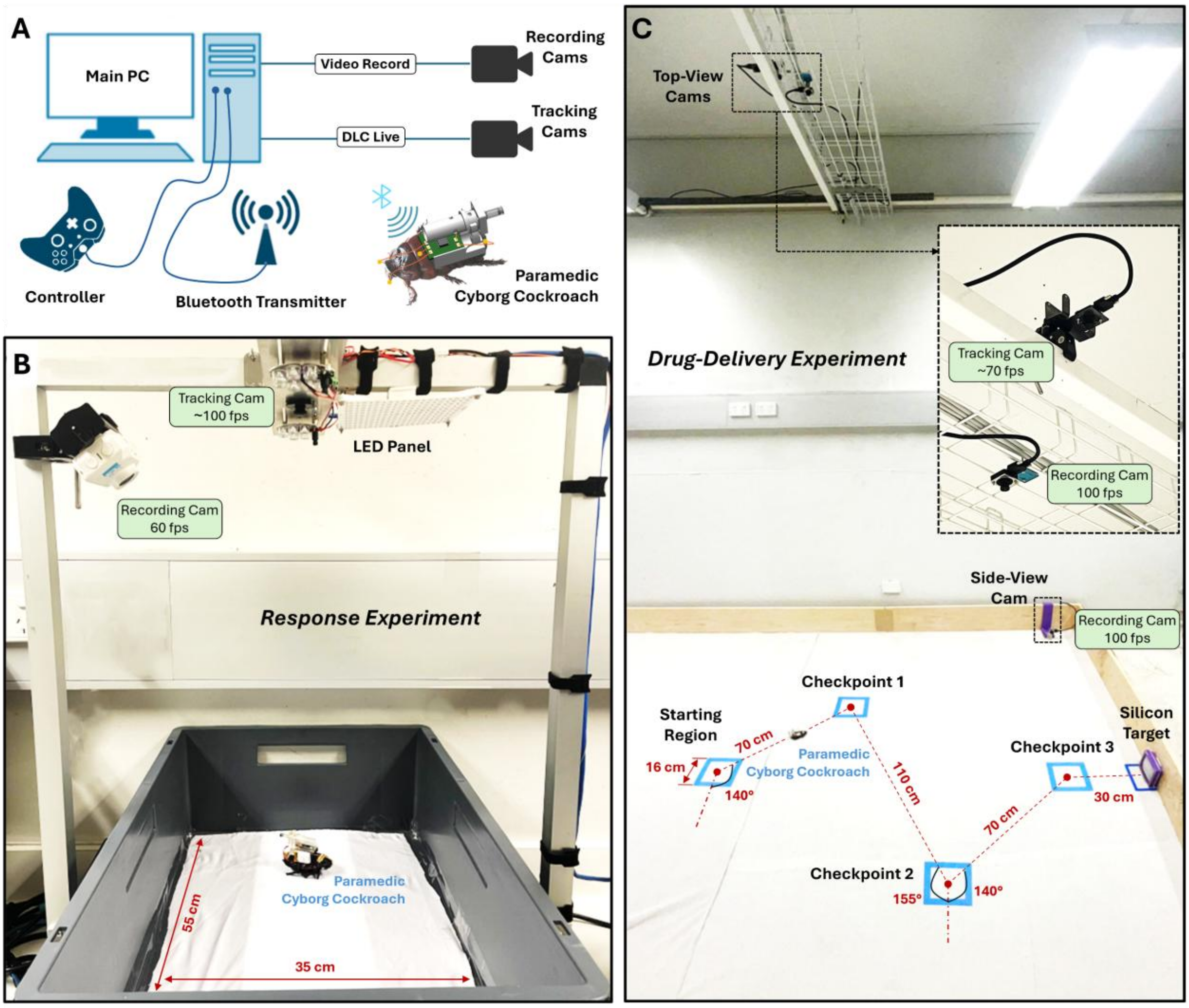

Figure S11. Experimental setup for investigating the performance of paramedic cyborg cockroaches. (A) General concept of experiment setup. Each experiment involved the manual control of Paraborg via Bluetooth communication. Locomotion was tracked and recorded using cameras positioned at multiple angles to capture both real-time position data and corresponding video. (B) Response experiment. Conducted in an enclosed arena to assess turning and acceleration responses of giant burrowing cockroaches under electrical stimulation applied to a unilateral antenna or both cerci respectively. (C) Drug-delivery experiment. Using the same configuration, this experiment validated Paraborg’s capability to navigate to designated checkpoints and perform in-situ drug delivery, demonstrating its potential as an insect-scale mobile robot for emergency scenarios.

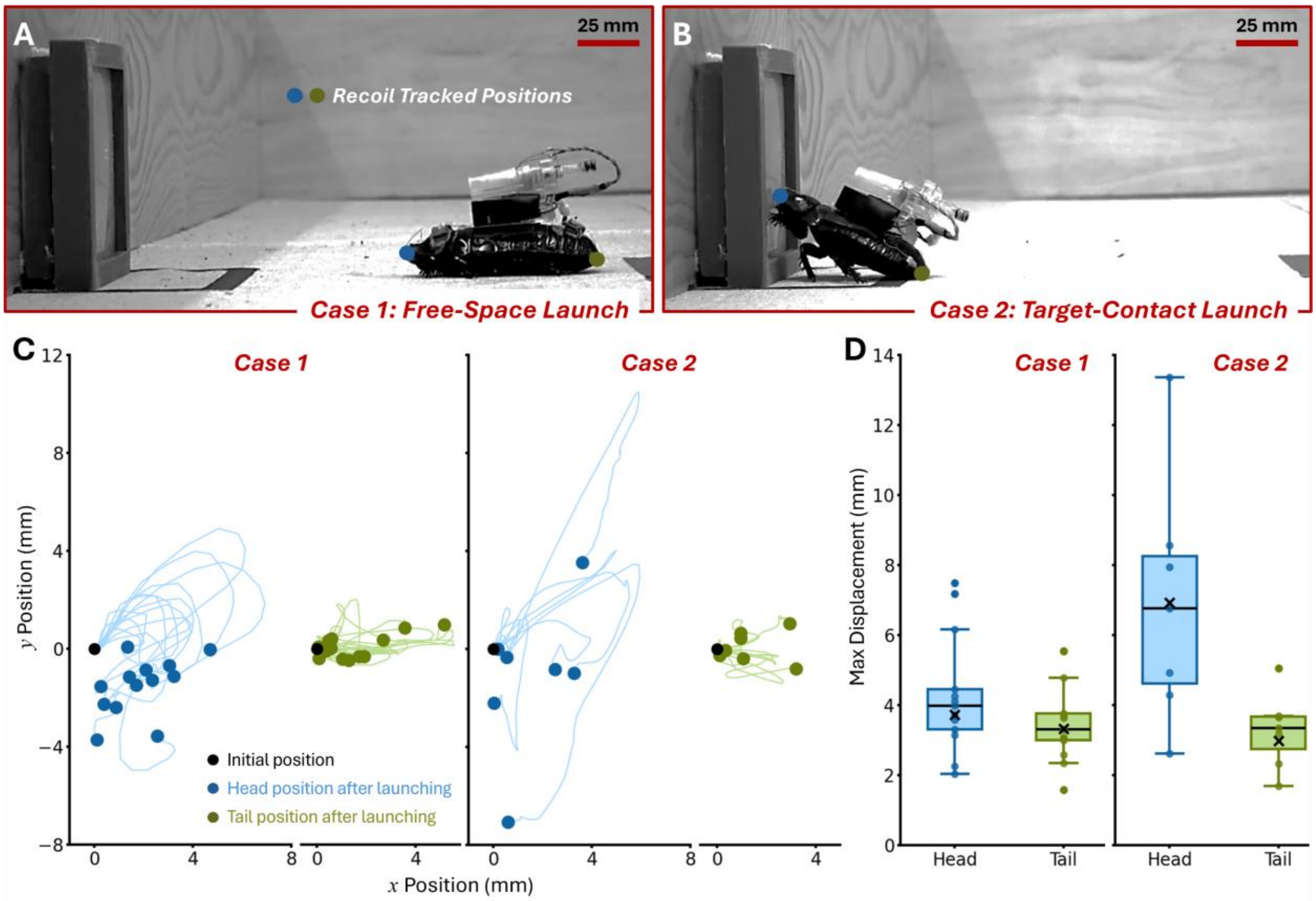

Figure S12. Recoil analysis of AIM launching with Paraborg. (A) Free-space launch. This scenario occurs when the cockroach enters the stabilizing state after passing the final checkpoint but remains at a random distance from the silicone target. Following immobilization via bilateral antenna stimulation, AIM is triggered without physical contact with the target. (B) Target-contact launch. In this case, the cockroach is in contact with the silicone target and is stabilized by electrical stimulation immediately prior to AIM triggering. (C) Body displacement during launch. Head and tail displacements were analyzed over a 1.5 s interval during the launching process, aggregated across all trials (15 free-space launches and 7 target-contact launches), to evaluate recoil effects. (D) Maximum displacement due to recoil. Maximum displacements of the head and tail relative to their initial positions (just before AIM triggering) were quantified from side-view tracking data and presented as boxplots (whiskers: 1.5× IQR, crosses indicate mean values). The results show that launching recoil induces a small bouncing displacement of the cockroach's head (~4 mm for free-space launch and ~7 mm for target-contact launch). In contrast, the rear body exhibits significantly smaller displacement, acting as a stabilizing constraint. In the target-contact condition, increased head displacement was attributed to an additional climbing response of the cockroach on the silicone surface during AIM activation.

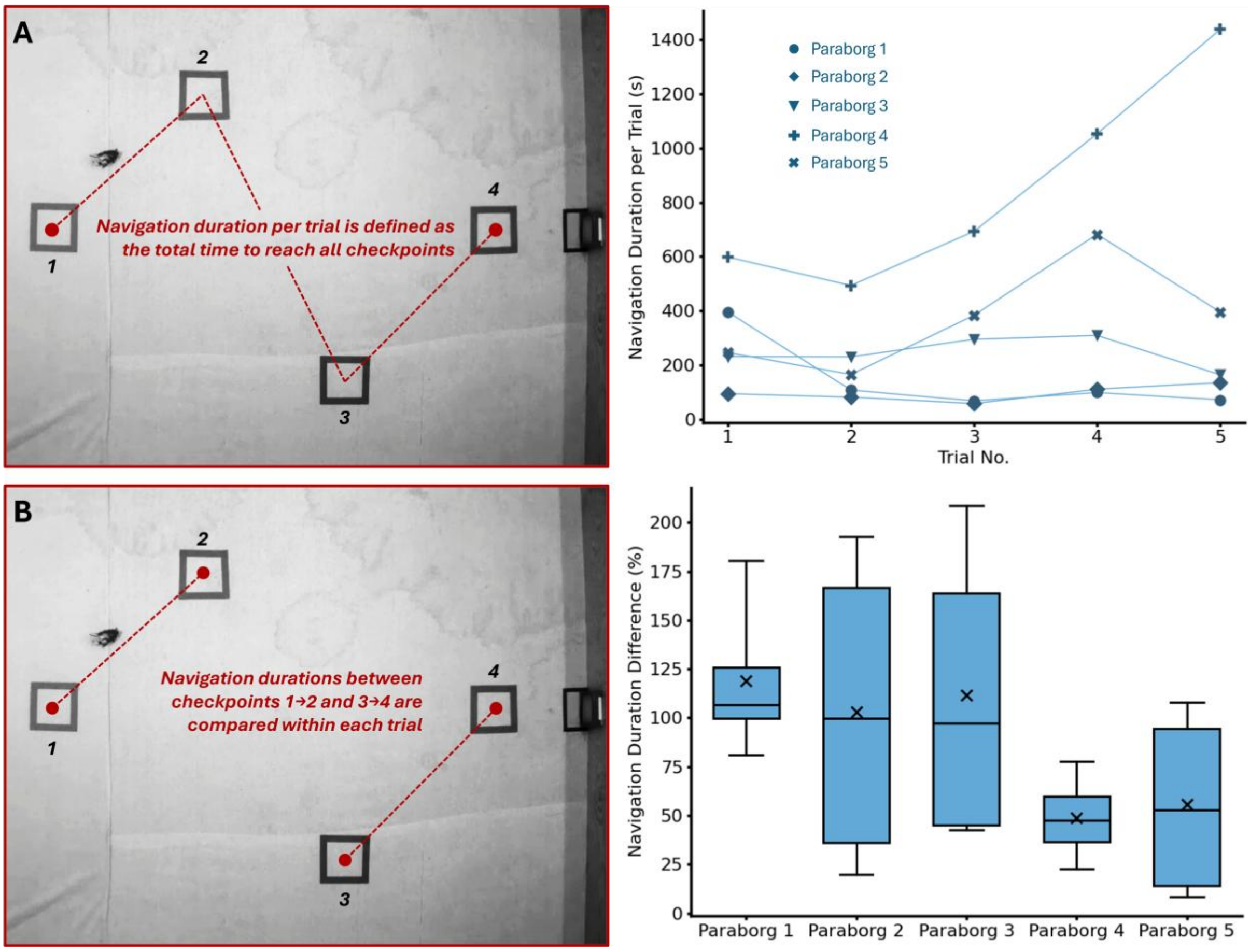

Figure S13. Timing analysis of Paraborg locomotion with AIM. (A) Navigation duration per trial with recovery periods. As locomotion control across four checkpoints achieved 100% success (*N* = 5 cockroaches, *n* = 25 trials), the total time required to traverse all checkpoints was analyzed for each trial (left). With at least 30 minutes of free-motion recovery between trials, 3 out of 5 Paraborgs maintained consistent navigation times below 5 minutes across trials (right). In contrast, Paraborg 4 exhibited increased durations from trials 2 to 4, while Paraborg 5 showed a progressive decline in speed, resulting in significantly longer navigation times. Overall, these results suggest that recovery periods can help restore locomotion performance and should be considered in future deployment and control strategies. (B) Navigation duration differences under continuous operation. To evaluate performance without recovery, we compared the time required to traverse two equal-distance segments within each trial (checkpoints 1 to 2 and 3 to 4; left). The relative increase in duration was calculated by normalizing the difference between these segments to the duration of the first segment (right). Across all 25 trials, increases in navigation time were observed, though with variability between trials. These results likely reflect cumulative fatigue and the constraints imposed by precise checkpoint navigation under manual control, leading to gradual performance decline over time.

Table S1. Various terrestrial insect-scale robots. The table summarizes the key characteristics of robots using two metrics: (i) payload-to-weight ratio and (ii) cost of transport (CoT). For systems reported in multiple publications, we considered the wireless configuration whenever available and selected the version demonstrating the most advanced performance. In total, 14 platforms from 18 publications published since 2018 were included, as they provided sufficient information for the selected metrics. For the payload-to-weight ratio, platforms with payload capacities suitable for integrating functional hardware are highlighted in bold, even though their ratios were not among the highest reported.

Robot name	Weight and Size	Linear Speed	Payload-to-Weight Ratio	Cost of Transport	Overview of Advantages and Limitations	Refs.
**Paraborg**	34 g<br>84 mm<br>37 mm<br>18 mm	34 mm/s	**0.76<br>(26 g payload)**	0.23<br>(4.0027 mW)	- Cyborg insects have been tested in practical terrains under fully wireless condition.<br>- Require no feedback systems for self-adaptability<br>- Onboard cameras and other sensors have been introduced	This study
Robot with mR-ES clutch (2026)	124 g<br>150 mm<br>120 mm<br>60 mm	-	-	34.65 mW	- No test with uneven terrains or obstructed environments<br>- No onboard sensors	(5)
HAMR (2025)	2.7 g<br>61 mm<br>37 mm<br>31 mm	518.5 mm/s	1.07<br>(2.9 g payload)	87.3<br>(1.2 W)	- Walking, climbing over low-inclined and smooth surfaces, jumping over obstacles<br>- Operated under tethered condition due to high voltage<br>- No onboard sensors	(6-10)
PiezoClimber (2024)	0.63 g<br>60 mm<br>42 mm<br>5 mm	84 mm/s	2.69<br>(1.7 g payload)	-	- Walking and climbing over vertical surfaces<br>- Operated under tethered condition due to high voltage<br>- No onboard sensors	(11)
**BHMbot-B (2024)**	1.89 g<br>20 mm<br>15 mm<br>15 mm	360 mm/s	**26.83<br>(50.7 g payload)**	105.1<br>(1.55 W)	- No test with uneven terrains or obstructed environments<br>- No onboard sensors	(12,13)
Microbot with morphing wheels (2024)	6.4 g<br>32 mm<br>37 mm<br>15 mm	152 mm/s	0.16<br>(2 g payload)	89	- Walking on terrains with moderate inclined level<br>- No onboard sensors	(14)
CLARI (2023)	2.59 g<br>44 mm<br>21 mm	28 mm/s	-	-	- Walking and adapting through narrow gaps<br>- Operated under tethered condition due to high voltage<br>- No onboard sensors	(15)
S^2worm (2022)	4.34 g<br>41 mm	274 mm/s	-	52.4<br>(610.5 mW)	- No test with uneven terrains or obstructed environments<br>- No onboard sensors	(16)
**Caterpillar-Inspired Robot (2022)**	1.7 g<br>40 mm<br>10 mm	12.3 mm/s	**5.88<br>(10 g payload)**	-	- Walking and climbing over vertical surfaces<br>- Operated under tethered condition due to high voltage	(17)
**Arthropod-Metamerism-Inspired Millirobot (2021)**	42.55 g<br>58 mm<br>44 mm<br>12 mm	516 mm/s	**4.7<br>(200 g payload)**	-	- Walking on terrains with moderate inclined level<br>- Operated under tethered condition due to high voltage<br>- No onboard sensors	(18)
Microrobot via Electromagnetically Induced Vibration (2021)	0.137 g<br>12.3 mm<br>20 mm<br>5 mm	232 mm/s	5.84<br>(0.8 g payload)	244<br>(76 mW)	- No test with uneven terrains or obstructed environments<br>- No onboard sensors	(19)
Rolling Microrobot (2020)	0.04 g<br>18 mm<br>8 mm<br>8 mm	27 mm/s	-	235.9<br>(2.5 mW)	- No test with uneven terrains or obstructed environments<br>- No onboard sensors	(20)
**Tribot (2019)**	10 g<br>45 mm<br>46 mm<br>47 mm	30 mm/s	**1.5<br>(15 g payload)**	3.7	- Jumping over moderate obstacles<br>- No onboard sensors	(21)
DEAnsect (2019)	0.19 g<br>40 mm	12 mm/s	5<br>(0.95 g payload)	5.3<br>(188 mW)	- No test with uneven terrains or obstructed environments<br>- No onboard sensors	(22)

- not reported
Green rows: Platforms not evaluated in practical environments
Red rows: Platforms relying on tethered power

Table S2. Detailed information on the integrated onboard hardware and corresponding mass. The table summarizes all components and their added mass for two Paraborg configurations: (i) Paraborg with AIM and (ii) Paraborg with an onboard camera.

Integrated Component	AIM-Carrying Paraborg	Cam-Carrying Paraborg	Mass	Potential for System Miniaturization
Resin mount	×		4 g	Yes
Main PCB, flexible pads and wires	×	×	5 g	No
Auto-injection module	×		6 g	Yes
Onboard camera (WiFi)		×	19 g	Yes
LiPo battery (3.7 V, 100 mAh)	×	×	2 g	No
**Total Added Mass**	17 g	26 g		

Table S3. Detailed information on AIM reuse during experiment. Across trials involving AIM launching and injections (Figures 3C, S9C), five AIM sets were reused for as many trials as possible prior to disposal. This table summarizes each AIM set, including the number of successful uses, components that required replacement (i.e., those that failed after the final successful trial), and the specific reasons for disposal.

AIM Set No.	Set 1	Set 2	Set 3	Set 4	Set 5
**Component Replacement**	-	Injector syringe<br>Acid chamber	Injector syringe<br>Launch tube	-	Injector syringe<br>Acid chamber
**No. of uses before disposal**	2	3	3	4	2
**Reason for Disposal**	Leakage during assembly of the injector syringe and acid chamber can initiate the reaction, resulting in unintended release of the liquid drug immediately after fabrication	After launch, the tube rebounded and fractured, preventing the injector from penetrating the silicone target; as a result, the needle bent	Similar to Set 1	Similar to Set 1	Similar to Set 1

- in Component Replacement: All AIM components from the earlier set were replaced

Table S4. Risk assessment of the complete drug delivery process using AIM. The table below summarizes these risks, including their descriptions, potential consequences, estimated severity and likelihood, as well as proposed mitigation strategies for future development.

Risk Category	Description of Risk	Potential Consequences	Severity	Likelihood	Mitigation Strategies (Future Work)
Device detachment after insertion	Syringe detaches after puncture with no securing structure on tissue	Loss of contact during delivery; incomplete or failed injection	Medium	Low	Temporary fixation/anchoring mechanisms, improve attachment design
Target movement	Subject movement following puncture due to pain or reflex	Syringe displacement or misalignment during delivery	High	Medium	Close-range launching and reduce puncture force, or use microneedle patches
Needle breakage	Mechanical loading from movement or detachment after insertion	Needle bending or fracture, retained fragments causing secondary injury	High	Medium	Reduce puncture force or use micro needle patches
Clinical incompatibility	Delivery time exceeds emergency treatment requirements	Not suitable for rapid-response drugs (e.g., epinephrine, antivenom)	Medium	High	Develop rapid delivery approaches, explore microneedle or alternative systems

3. Supplementary Movies

Movie S1. Locomotion control of paramedic cyborg cockroach via electrical stimulation. Locomotor responses of terrestrial cyborg insects to electrical stimulation at sensory organs were demonstrated to verify effective terrestrial maneuverability and reliable controllability when employing living insects as organic mobile robot platforms. Two representative graded locomotor outputs were observed: (i) left/right turning in response to electrical stimulation at the right/left antenna, and (ii) acceleration in response to electrical stimulation at both cerci.

Movie S2. Effect mounted payload on cockroach locomotion performance. To verify that carrying a backpack does not significantly affect the overall locomotion performance of live cockroaches, two sets of experiments were conducted. First, locomotion of loaded and unloaded cockroaches was compared under three behavioral conditions: free walking, wall-following, and corner turning. Second, climbing performance was evaluated across three configurations: (i) an unloaded cockroach, (ii) Paraborg equipped with AIM, and (iii) Paraborg with an onboard camera.

Movie S3. Demonstration of the auto-injection mechanism (AIM). The operating principle of the Auto-Injection Mechanism (AIM) was experimentally validated through two sequential processes: (i) injector deployment via proactive triggering and (ii) autonomous liquid-drug delivery following successful target penetration. To evaluate performance across different target conditions, AIM was tested on both a highly compliant silicone layer and a pork belly specimen representing real biological tissue. Successful operation was demonstrated in both cases, confirming the feasibility of autonomous injection into soft tissue targets.

Movie S4. Successful in-situ drug delivery at close range by single Paraborg. Reliable needle-based drug delivery by a paramedic cyborg cockroach was demonstrated through a successful experimental trial in which the insect navigated through three checkpoints, approached the effective launch range, and then performed in-situ drug delivery with the AIM. During the trial, the Paraborg employed not only fundamental control strategies, i.e., left/right turning and acceleration, but also a U-turn counter to overcome habituation at sensory organs and ensure accurate positioning by orienting toward the assigned target.

Movie S5. Real-time visualization using onboard camera in Paraborg. With strong load-carrying capacity, the cyborg cockroach was capable of carrying an onboard camera for real-time visualization, which supported the human controller in effectively exploring complex environments or identifying trapped victims after natural disasters. During locomotion with the onboard camera, the moving speed and navigation tendency were confirmed to be unaffected by the additional weight and height of the integrated hardware.

Movie S6. Teamwork concept for dispersed Paraborg members in field deployment. In-situ drug delivery by multiple Paraborgs was confirmed to be feasible in the first practical scenario, in which two functional cyborgs were dispersed. After identifying the assigned target, the observer cyborg was stabilized at a position where the onboard camera could capture a panoramic view of the surrounding area. Meanwhile, the drug-delivery cyborg was navigated toward the camera-integrated cyborg until it entered the onboard camera's view, after which it performed needle-based drug delivery under visual supervision.

Movie S7. Teamwork concept for co-located Paraborg members. The dual-agent operation concept underlying future Paraborg swarm implementations was demonstrated using two Paraborgs. During navigation of the drug-delivery cyborg through a series of assigned checkpoints, stable co-localization between the two agents was maintained, enabling

simultaneous target search and real-time supervision by the camera-equipped observer cyborg. Furthermore, the dual-agent Paraborg concept was validated through navigation trials involving obstacles that induced temporary loss of line of sight, demonstrating the system's ability to maintain coordinated operation in challenging environments. These results highlight the potential of the approach for deployment in real-world search-and-rescue missions and inspection tasks within confined, cluttered, or otherwise hard-to-reach areas.

References

1. “Cyborg Cockroach Sparks Ethics Debate.” Accessed: Jun. 16, 2026. Available: https://www.science.org/content/article/cyborg-cockroach-sparks-ethics-debate
2. C. J. Perry, A. B. Barron, and L. Chittka, “The frontiers of insect cognition,” Curr. Opin. Behav. Sci., vol. 16, pp. 111–118, Aug. 2017, doi: 10.1016/j.cobeha.2017.05.011.
3. H. Tiffin, “Do Insects Feel Pain?,” Anim. Stud. J., vol. 5, no. 1, Jun. 2016, Accessed: Jun. 16, 2026. Available: https://www.uowoajournals.org/asj/article/id/232/
4. K. Mamak, “Whether to Save a Robot or a Human: On the Ethical and Legal Limits of Protections for Robots,” Front. Robot. AI, vol. 8, Jul. 2021, doi: 10.3389/frobt.2021.712427.
5. J. Lee, A. Firouzeh, S. An, P. Lee, and K.-J. Cho, “A Miniature Rotary Electrostatic Clutch for Assigning Multi-Degrees of Freedom to Insect-Scale Robots,” Adv. Intell. Syst., vol. 8, no. 3, p. 2500625, 2026, doi: 10.1002/aisy.202500625.
6. F. Ramirez Serrano, N. P. Hyun, E. Steinhardt, P.-L. Lechère, and R. J. Wood, “A springtail-inspired multimodal walking-jumping microrobot,” Sci. Robot., vol. 10, no. 99, p. eadp7854, Feb. 2025, doi: 10.1126/scirobotics.adp7854.
7. P. E. Schiebel, J. Shum, H. Cerbone, and R. J. Wood, “An insect-scale robot reveals the effects of different body dynamics regimes during open-loop running in feature-laden terrain,” Bioinspir. Biomim., vol. 17, no. 2, p. 026006, Feb. 2022, doi: 10.1088/1748-3190/ac3f7d.
8. K. Jayaram, J. Shum, S. Castellanos, E. F. Helbling, and R. J. Wood, “Scaling down an insect-size microrobot, HAMR-VI into HAMR-Jr,” in 2020 IEEE International Conference on Robotics and Automation (ICRA), May 2020, pp. 10305–10311. doi: 10.1109/ICRA40945.2020.9197436.
9. B. Goldberg, N. Doshi, and R. J. Wood, “High speed trajectory control using an experimental maneuverability model for an insect-scale legged robot,” in 2017 IEEE International Conference on Robotics and Automation (ICRA), May 2017, pp. 3538–3545. doi: 10.1109/ICRA.2017.7989405.
10. B. F. Seitz et al., “Bio-inspired mechanisms for inclined locomotion in a legged insect-scale robot,” in 2014 IEEE International Conference on Robotics and Biomimetics (ROBIO 2014), Dec. 2014, pp. 791–796. doi: 10.1109/ROBIO.2014.7090428.
11. M. Zheng, D. Wang, D. Zhu, S. Cao, X. Wang, and M. Zhang, “PiezoClimber: Versatile and Self-Transitional Climbing Soft Robot with Bioinspired Highly Directional Footpads,” Adv. Funct. Mater., vol. 34, no. 6, p. 2308384, 2024, doi: 10.1002/adfm.202308384.
12. X. Yu et al., “Forward and backward control of an ultrafast millimeter-scale microrobot via vibration mode transition,” Sci. Adv., vol. 10, no. 43, p. eadr1607, Oct. 2024, doi: 10.1126/sciadv.adr1607.
13. Z. Liu et al., “A wireless controlled robotic insect with ultrafast untethered running speeds,” Nat. Commun., vol. 15, no. 1, p. 3815, May 2024, doi: 10.1038/s41467-024-47812-5.
14. Y. Lai et al., “An agile multimodal microrobot with architected passively morphing wheels,” Sci. Adv., vol. 10, no. 51, p. eadp1176, Dec. 2024, doi: 10.1126/sciadv.adp1176.
15. H. Kabutz and K. Jayaram, “Design of CLARI: A Miniature Modular Origami Passive Shape-Morphing Robot,” Adv. Intell. Syst., vol. 5, no. 12, p. 2300181, 2023, doi: 10.1002/aisy.202300181.

16. Y. Liu et al., “S2worm: A Fast-Moving Untethered Insect-Scale Robot With 2-DoF Transmission Mechanism,” IEEE Robot. Autom. Lett., vol. 7, no. 3, pp. 6758–6765, Jul. 2022, doi: 10.1109/LRA.2022.3176435.
17. M. M. Khalil and T. Mashimo, “Caterpillar-Inspired Insect-Scale Climbing Robot Using Dry Adhesives,” IEEE Robot. Autom. Lett., vol. 7, no. 3, pp. 7628–7635, Jul. 2022, doi: 10.1109/LRA.2022.3183897.
18. Y. Liu et al., “Arthropod-Metamerism-Inspired Resonant Piezoelectric Millirobot,” Adv. Intell. Syst., vol. 3, no. 8, p. 2100015, 2021, doi: 10.1002/aisy.202100015.
19. X. Liu et al., “Moving Mechanism of a High-speed Insect-scale Microrobot via Electromagnetically Induced Vibration,” J. Bionic Eng., vol. 18, no. 3, pp. 662–673, May 2021, doi: 10.1007/s42235-021-0037-8.
20. P. Bhushan and C. Tomlin, “An Insect-Scale Self-Sufficient Rolling Microrobot,” IEEE Robot. Autom. Lett., vol. 5, no. 1, pp. 167–172, Jan. 2020, doi: 10.1109/LRA.2019.2949735.
21. Z. Zhakypov, K. Mori, K. Hosoda, and J. Paik, “Designing minimal and scalable insect-inspired multi-locomotion millirobots,” Nature, vol. 571, no. 7765, pp. 381–386, Jul. 2019, doi: 10.1038/s41586-019-1388-8.
22. X. Ji et al., “An autonomous untethered fast soft robotic insect driven by low-voltage dielectric elastomer actuators,” Sci. Robot., vol. 4, no. 37, p. eaaz6451, Dec. 2019, doi: 10.1126/scirobotics.aaz6451.
23. H. Min et al., “Stiffness-tunable velvet worm–inspired soft adhesive robot,” Sci. Adv., vol. 10, no. 47, p. eadp8260, Nov. 2024, doi: 10.1126/sciadv.adp8260.
24. K. Johnson, Z. Englhardt, V. Arroyos, D. Yin, S. Patel, and V. Iyer, “MilliMobile: An Autonomous Battery-free Wireless Microrobot,” in Proceedings of the 29th Annual International Conference on Mobile Computing and Networking, in ACM MobiCom ’23. New York, NY, USA: Association for Computing Machinery, Oct. 2023, pp. 1–16. doi: 10.1145/3570361.3613304.
25. S. Wu, Y. Hong, Y. Zhao, J. Yin, and Y. Zhu, “Caterpillar-inspired soft crawling robot with distributed programmable thermal actuation,” Sci. Adv., vol. 9, no. 12, p. eadf8014, Mar. 2023, doi: 10.1126/sciadv.adf8014.
26. Y. Kim et al., “Remote control of muscle-driven miniature robots with battery-free wireless optoelectronics,” Sci. Robot., vol. 8, no. 74, p. eadd1053, Jan. 2023, doi: 10.1126/scirobotics.add1053.
27. R. M. Bena, X.-T. Nguyen, A. A. Calderón, A. Rigo, and N. O. Pérez-Arancibia, “SMARTI: A 60-mg Steerable Robot Driven by High-Frequency Shape-Memory Alloy Actuation,” IEEE Robot. Autom. Lett., vol. 6, no. 4, pp. 8173–8180, Oct. 2021, doi: 10.1109/LRA.2021.3070246.
28. X. Dong, C. Tang, S. Jiang, Q. Shao, and H. Zhao, “Increasing the Payload and Terrain Adaptivity of an Untethered Crawling Robot Via Soft-Rigid Coupled Linear Actuators,” IEEE Robot. Autom. Lett., vol. 6, no. 2, pp. 2405–2412, Apr. 2021, doi: 10.1109/LRA.2021.3061342.
29. X. Yang, L. Chang, and N. O. Pérez-Arancibia, “An 88-milligram insect-scale autonomous crawling robot driven by a catalytic artificial muscle,” Sci. Robot., vol. 5, no. 45, p. eaba0015, Aug. 2020, doi: 10.1126/scirobotics.aba0015.
30. Y. Wu et al., “Insect-scale fast moving and ultrarobust soft robot,” Sci. Robot., vol. 4, no. 32, p. eaax1594, Jul. 2019, doi: 10.1126/scirobotics.aax1594.
31. J. Cao et al., “Untethered soft robot capable of stable locomotion using soft electrostatic actuators,” Extreme Mech. Lett., vol. 21, pp. 9–16, May 2018, doi: 10.1016/j.eml.2018.02.004.
